# Gene Expression Analysis Reveals the Cell Cycle and Kinetochore Genes Participating in Ischemia Reperfusion Injury and Early Development in Kidney

**DOI:** 10.1371/journal.pone.0025679

**Published:** 2011-09-28

**Authors:** Tae-Min Kim, Victoria Ramírez, Jonatan Barrera-Chimal, Norma A. Bobadilla, Peter J. Park, Vishal S. Vaidya

**Affiliations:** 1 Center for Biomedical Informatics, Harvard Medical School, Boston, Massachusetts, United States of America; 2 Renal Division, Department of Medicine, Brigham and Women's Hospital, Harvard Medical School, Boston, Massachusetts, United States of America; 3 Molecular Physiology Unit, Instituto de Investigaciones Biomédicas, Universidad Nacional Autónoma de México, Mexico City, Mexico; 4 Departamento de Nefrología y Metabolismo Mineral, Instituto Nacional de Ciencias Médicas y Nutrición Salvador Zúbiran, Mexico City, Mexico; 5 Department of Environmental Health, Harvard School of Public Health, Boston, Massachusetts, United States of America; The Centre for Research and Technology, Hellas, Greece

## Abstract

**Background:**

The molecular mechanisms that mediate the ischemia-reperfusion (I/R) injury in kidney are not completely understood. It is also largely unknown whether such mechanisms overlap with those governing the early development of kidney.

**Methodology/Principal Findings:**

We performed gene expression analysis to investigate the transcriptome changes during regeneration after I/R injury in the rat (0 hr, 6 hr, 24 hr, and 120 hr after reperfusion) and early development of mouse kidney (embryonic day 16 p.c. and postnatal 1 and 7 day). Pathway analysis revealed a wide spectrum of molecular functions that may participate in the regeneration and developmental processes of kidney as well as the functional association between them. While the genes associated with cell cycle, immunity, inflammation, and apoptosis were globally activated during the regeneration after I/R injury, the genes encoding various transporters and metabolic enzymes were down-regulated. We also observed that these injury-associated molecular functions largely overlap with those of early kidney development. In particular, the up-regulation of kinases and kinesins with roles in cell division was common during regeneration and early developmental kidney as validated by real-time PCR and immunohistochemistry.

**Conclusions:**

In addition to the candidate genes whose up-regulation constitutes an overlapping expression signature between kidney regeneration and development, this study lays a foundation for studying the functional relationship between two biological processes.

## Introduction

Acute kidney injury represents a common clinical problem leading to high rates of morbidity and mortality [1]. Identification of accurate diagnostic markers or effective targets for therapeutic intervention is challenging and it would benefit from a comprehensive understanding about the molecular programs governing renal injury-regeneration processes [2], [3]. Kidney regeneration after ischemic injury represents a complicated but highly coordinated process, which involves a diverse set of molecular functions. For example, animal studies using microarray-based expression analysis revealed that various molecular functions (e.g., cell cycle, inflammation and apoptosis) were perturbed in ischemic-reperfusion (I/R) injury [4]–[7]. It has also been proposed that the transcriptional reprogramming induced by I/R injury may recapitulate those of kidney organogenesis in which the kidney injury induces the re-expression of various nephrogenic genes such as Vimentin, Pax-2 and Bmp-7 [8]–[10]. Considering that these two biological processes (kidney regeneration and development) require tightly regulated cellular processes that control cell proliferation and differentiation, a comparison between the transcriptional programs associated with these processes may provide a better understanding about the underlying molecular mechanisms, as exemplified in other organogenesis model (e.g., liver) [11], [12].

In this study, we performed microarray-based gene expression analysis to investigate changes in the transcriptome during kidney I/R injury and early development using animal models. Pathway analysis of the transcriptome changes revealed that a wide spectrum of molecular functions may be involved in these biological processes. A detailed examination on the relationship between regeneration- and development-related genes in terms of their enrichment to known functional gene categories further showed that some molecular functions are common to kidney regeneration and development. Among the shared functions, we focused on a number of cell cycle and kinetochore genes that were over-expressed during I/R injury and embryonic kidneys with experimental verification.

## Results

### Adult kidney injury model using bilateral ischemia-reperfusion injury

**Figure 1:**
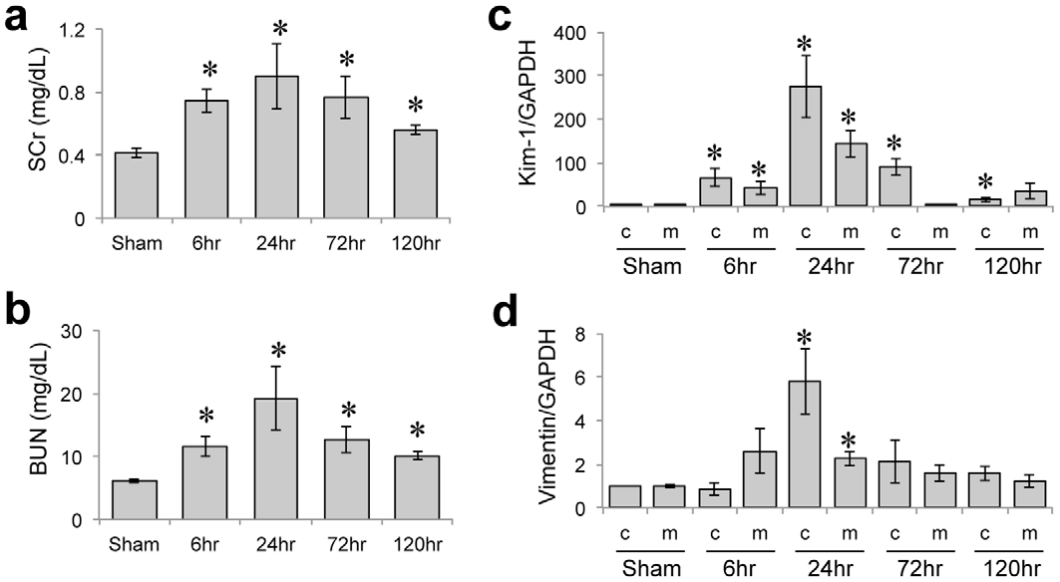
Temporal profiles of kidney biomarkers in ischemic-reperfusion kidney model. **A.** SCr levels are measured after 6 hr, 24 hr, 72 hr, and 120 hr of reperfusion after I/R injury in rat. Significant difference of SCr level compared to Sham is shown with asterisk (*P*<0.05). **B.** The temporal changes in BUN levels are shown. Renal mRNA levels of Kim-1 (**C**) and Vimentin (**D**) are shown for cortex and medulla. The mRNA levels were normalized using GAPDH as control gene (denoted as c and m, respectively). * represents *P*<0.05 as determined by one way ANOVA in comparison with sham rats.

**Figure 2:**
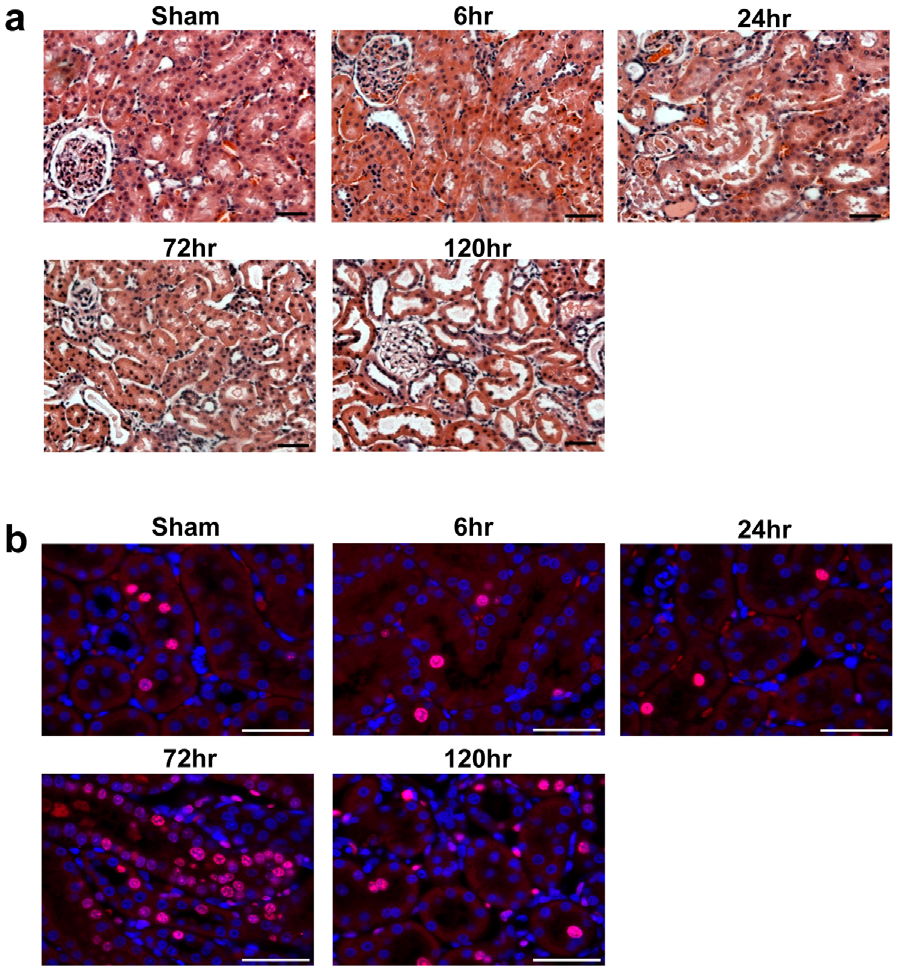
Histological and immunohistochemical examination of kidney tissue injury and repair following I/R injury. Representative formalin fixed paraffin embedded kidney tissue sections of sham male Wistar rats and those undergone 20 min bilateral ischemia reperfusion over time stained by (**A**) Hematoxylin and Eosin (40× magnification) and (**B**) Ki-67 (60× magnification). *n* = 5/group. Bar represents 50 µm.

Kidney injury was induced by 20 minutes of bilateral renal ischemia followed by reperfusion that resulted in kidney dysfunction, characterized by elevation of serum creatinine (SCr) and blood urea nitrogen (BUN), (Figure 1A and 1B) at 24 hr of reperfusion followed by a recovery at 120 hr. Kidney Injury Molecule-1 (Kim-1) mRNA levels , recently identified as sensitive indicator in monitoring tubular kidney injury [3] and Vimentin mRNA levels, a marker of mesenchymal phenotype and dedifferentiation of tubular cells [13] also showed peak expression at 24 hr after injury while the expression decreased to control levels after 120 hr (Figure 1C and 1D). The histopathological examination showed typical lesions caused by renal ischemia at 24 hr characterized by proximal tubular necrosis and apoptosis that resulted in substantial loss in epithelial brush border and cell debris accumulation in luminal area at 24 hr with a structural and functional recovery by 120 hr due to an efficient kidney regeneration response (Figure 2A). The immunohistochemistry of Ki-67 showed a marked staining level at 72 hr and 120 hr after I/R injury (Figure 2B), which indicates the enhanced cellular proliferation during these regenerative time points.

### Animal model for embryonic kidney development and comparative gene expression profiling

To take into account the differential expression pattern between renal cortex and medulla, we isolated total RNA from rat renal cortices and medullas and performed gene expression profiling at 6 hr, 24 hr and 120 hr which correspond to early, intermediate and late-regeneration phase after I/R injury (*n* = 3 for each). To explore the expression profiles of kidney development, we also collected RNA from mouse kidney at embryonic day 16 postcoitum or p.c. (E16d) and at postnatal day 1 and 7 (PN1d and PN7d; *n* = 5 for each), respectively. We selected E16d since this time point is critical in early kidney development (e.g., distinction of cortex versus medulla and the beginning of differentiation of functional proximal tubule) and PN1d and PN7d represent the immediate and early postnatal period, respectively.

**Figure 3:**
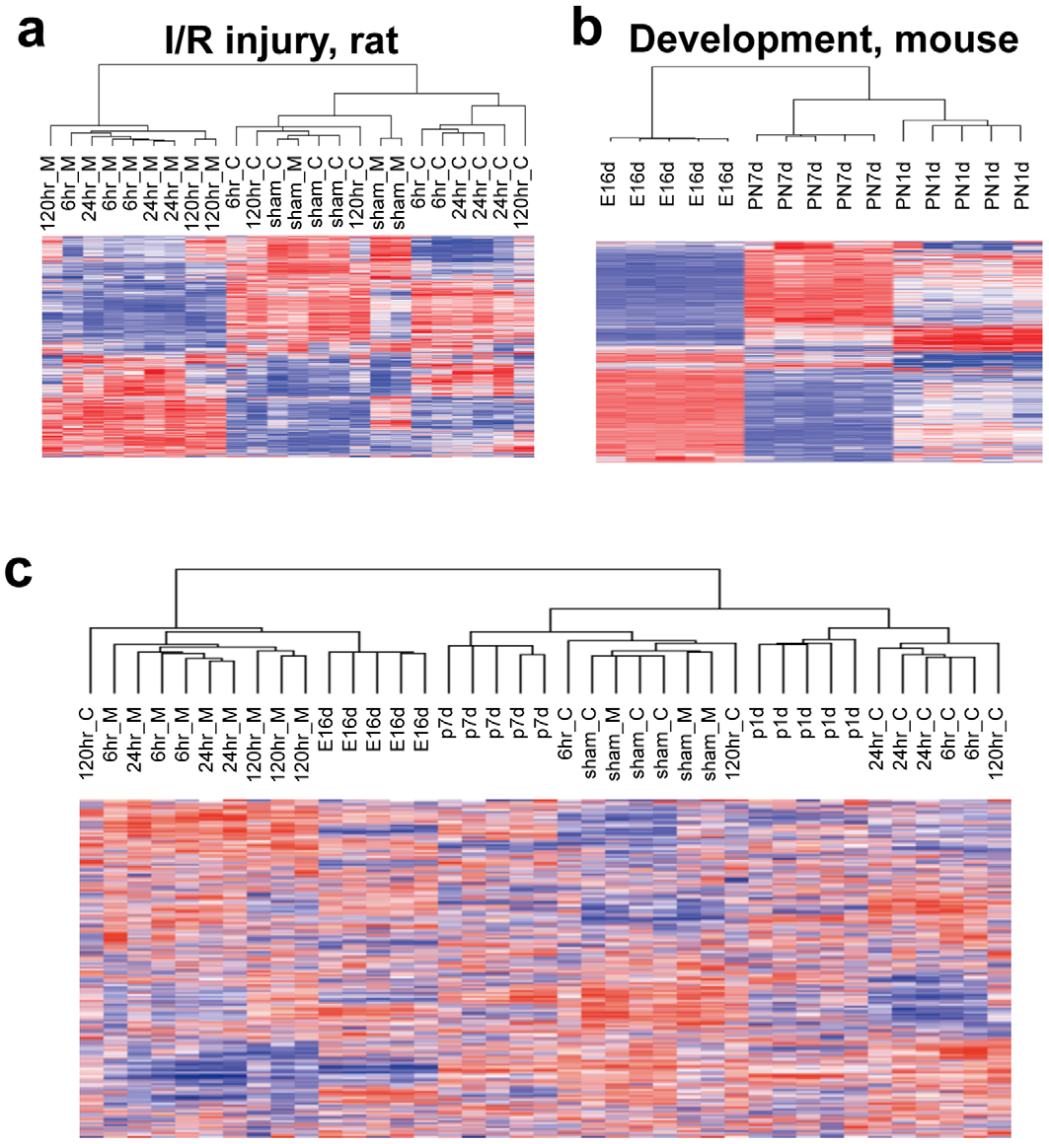
Hierarchical clustering of kidney regeneration after ischemic injury and development. **A.** Hierarchical clustering of top variable 2000 genes segregates the expression profiles of renal cortex (C) and medulla (M) after ischemic injury while sham samples are also distinguished from injury-associated profiles. **B.** The expression profiles of fetal mouse kidney (E16d) are clearly distinguished from those of postnatal kidney (PN1d and PN7d) by hierarchical clustering. C. The rat I/R injury- and mouse development expression profiles were merged and hierarchically clustered for top variable 2000 orthologous genes.

The hierarchical clustering of highly variable genes showed that the expression profiles of renal cortex and medulla are largely distinguishable from each other and the profiles from kidneys subjected to I/R injury are also distinguishable from those of sham (Figure 3A). The hierarchical clustering also clearly separated the expression profiles of embryonic mouse kidney from those of postnatal kidneys (Figure 3B). We also combined the expression profiles of rat I/R injury and mouse developmental kidneys and performed hierarchical clustering using highly variable orthologous genes (Figure 3C). The segregating pattern observed when two species were separately analyzed (e.g., three main clusters in each profile) was preserved in the combined analysis. The clustering of merged dataset further reveals the expression similarities between two species such as sham (rat) and PN7d (mouse) as well as E16d (mouse)-ischemic medulla (rat) and PN1d (mouse)-ischemic cortex (rat).

**Figure 4:**
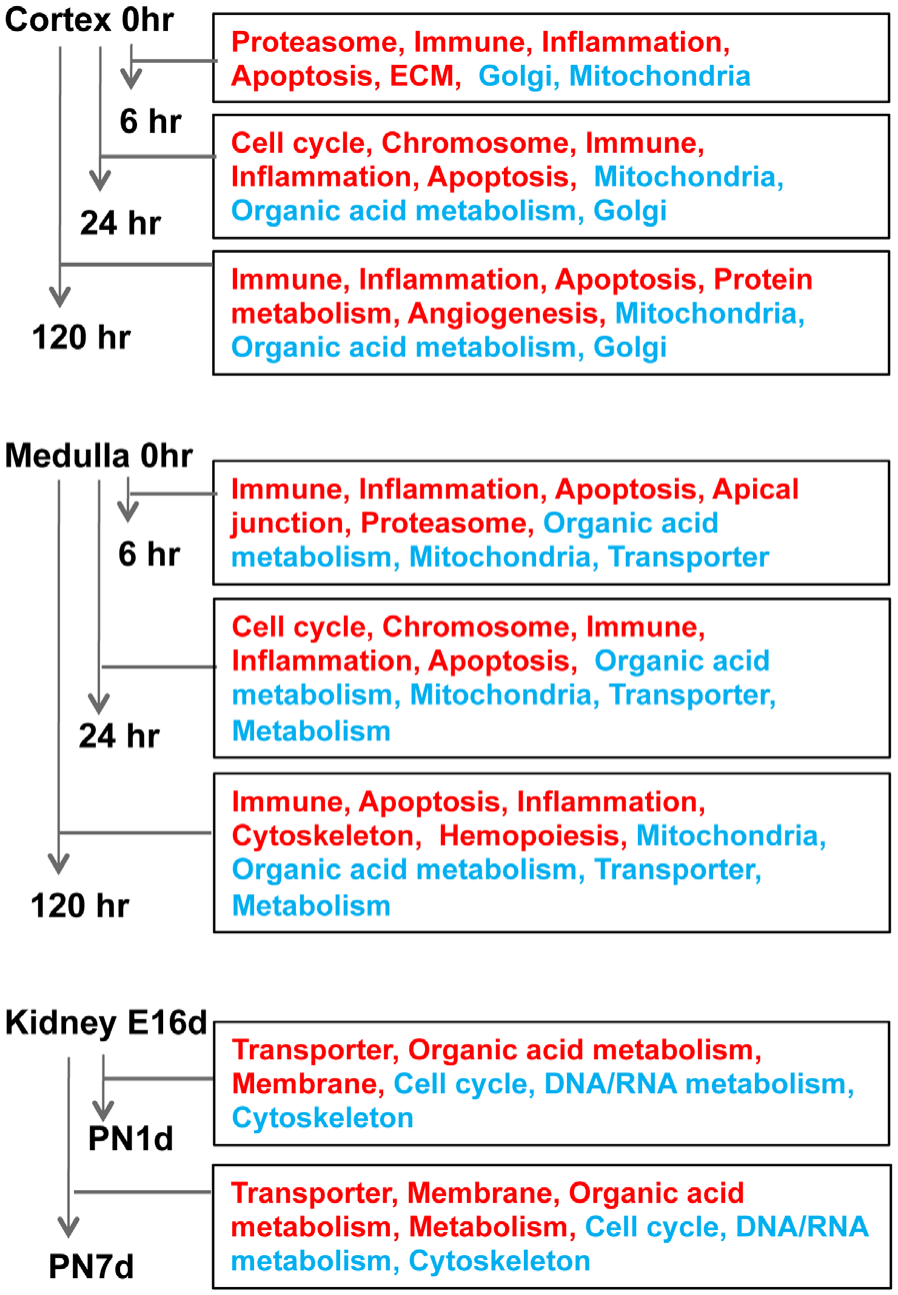
Pathway analysis of expression changes associated with kidney regeneration after ischemic injury and development. Pathway analysis shows the significantly up- and down-regulated GO functional categories (red- and blue-coded in the corresponding rectangles, respectively) in each time point measured after ischemic induction (cortex and medulla of rat kidney) or in the corresponding developmental time points (mouse kidney). Only representational functional categories are shown from a list of significantly enriched GO categories. Full list of observed GO categories are available in Tables S1,S2,S3.

Next, we performed pathway analysis to explore the coordinated expression changes of functionally related genes during kidney I/R injury and development. Parametric analysis of gene expression (PAGE) [14] was used to measure the enrichment of Gene Ontology (GO) functional categories [15] between two phenotypes (e.g., sham versus 6-hr in renal cortex). We collected GO annotations with significant enrichment (*P*<0.05, Bonferroni adjusted) across eight comparisons, i.e., six for I/R injury (6 hr, 24 hr and 120 hr for cortex and medulla; each compared to sham) and two for development (PN1d and PN7d; each compared to E16d). Figure 4 shows the representative functional categories in each comparison (full lists of significant functions are available in Tables S1,S2,S3). We observed that the molecular functions representing immunity, inflammation and apoptosis are globally up-regulated across all time points measured after I/R injury (6 hr, 24 hr and 120 hr) in cortex and medulla indicating that these functions are characteristic transcriptional features of kidney regeneration after ischemic injury. In addition, the molecular functions associated with metabolism or ion transport are commonly down-regulated during kidney regeneration.

We observed that some molecular functions are observed both during kidney regeneration and development. For example, cell cycle and chromosome-related functions are up-regulated at 24 hr after I/R injury, while down-regulated in postnatal kidneys compared to embryonic ones. Also, the up-regulated functions such as ion transporters or metabolism in postnatal kidney (or down-regulated in embryonic kidney) largely overlap with those observed in ischemic-regeneration profiles.

**Figure 5:**
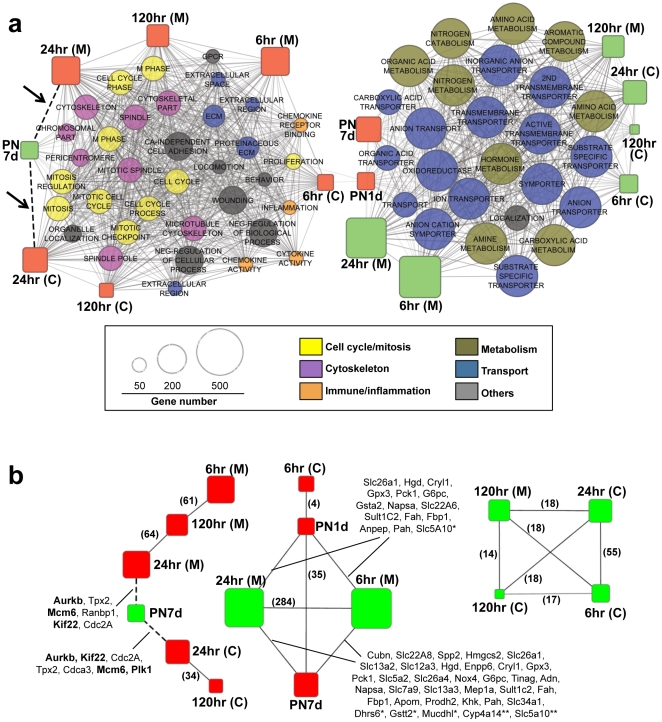
Functional association network. **A.** The differentially expressed genes during regeneration or development of kidney (the up- and down-regulated genes as red and green rectangles, respectively) and GO categories (circles) are shown as nodes. The significant enrichment between two gene sets is shown as edge between two corresponding nodes. The size of node is proportional to the gene size and GO categories were classified into six major functions as shown in the color scheme. Two dashed lines with arrows represent the significant association between PN7d (genes down-regulated compared to E16d) and 24 hr I/R (genes up-regulated compared to sham). **B.** The significant associations between gene sets are shown after removing GO categories. The overlapping genes are shown for pairs between I/R injury and development. Experimentally validated four genes are bold. * and ** represent the genes only observed with 6 hr/medulla and 24 hr/medulla, respectively. Number of overlapping genes are shown in parenthesis for significant pairs between I/R injury gene sets.

We further constructed a functional network comprising the up- and down-regulated genes in the two processes and their enriched GO categories as nodes (Figure 5A). Their associations (significant gene overlap between gene sets) are illustrated as edges. The subnetwork on the left (Figure 5A) includes six up-regulated gene sets during kidney ischemic-regeneration (red rectangles) and one down-regulated gene set (one green rectangle) in postnatal kidney compared to embryonic kidney. This subnetwork also includes multiple GO categories representing cell cycle/mitosis, cytoskeleton and immune/inflammation. The other subnetwork is largely associated with functional categories of transport/membrane and metabolism and composed of regeneration-down-regulated and postnatal-up-regulated genes (Figure 5A, right). Thus, our pathway analysis reveals that the differentially expressed genes in both regenerative and developmental time points share common functional gene sets, highlighting the similarity in the two processes.

When the GO nodes are removed (the differentially expressed genes in regeneration or development are compared directly), a substantial overlap among datasets becomes clearer (Figure 5B). Among the significant gene overlap between differentially expressed genes, we focused on those between regenerative- and developmental profiles and their overlapping genes (listed in Figure 5B). For example, we observed significant gene overlap between PN7d (down-regulated) and 24 hr I/R (up-regulated; both in cortex and medulla) and the overlapping genes largely represent kinase and kinetochore-encoding genes. The up-regulated genes in PN1d and PN7d were enriched in the down-regulated genes of 6 hr and 24 hr after I/R injury in medulla. These genes largely represent membrane-associated or ion- transport molecules (e.g., solute carrier gene families, Nox4, Mep1A, Apom, and Anpep). The functional annotations of overlapping genes are consistent with the enriched functional categories between two biological processes also highlighting the overlapping genes as candidates for molecular validation.

### Confirmation of overlapping genes

For experimental validation, we focused on the overlapping genes between those down-regulated at postnatal 7 day (PN7d) kidney and those up-regulated at 24 hr after I/R injury in renal cortex and medulla (Figure 5A; two edges indicated by arrows). Eight genes were observed in either edge. Aurkb, Kif22 (or Kid), Cdc2A (or Cdk1), Tpx2, and Mcm6 were observed in both edges while Cdca3, Plk1 (in medulla only) and Ranbp1 (in cortex only) were observed in single edge. These genes are primarily associated with celldivision functions as essential components of kinetochore.

**Figure 6:**
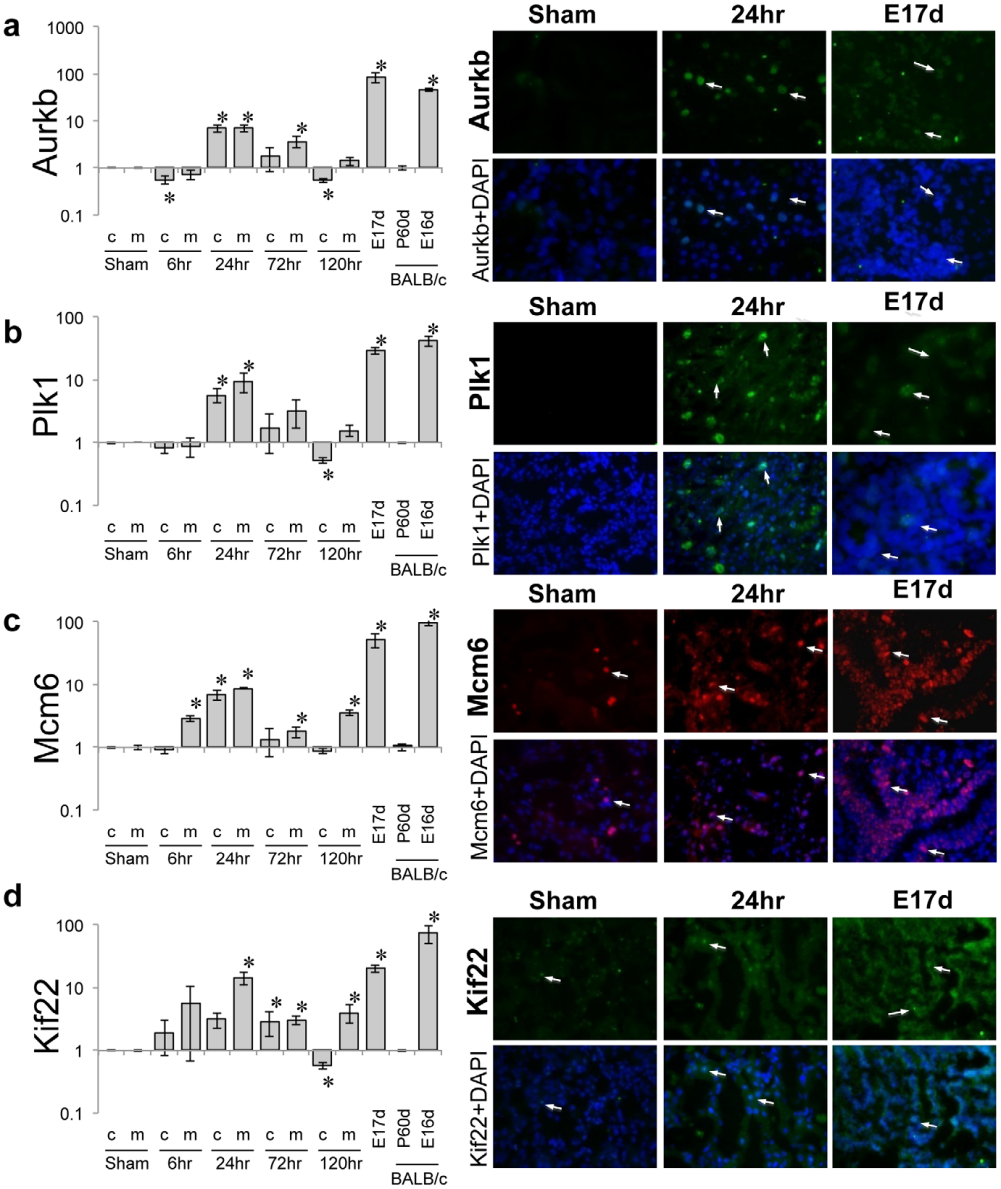
Quantitative mRNA levels. **A.** mRNA levels of Aurkb were measured by real-time quantitative PCR in rat and mouse kidney (left). Rat kidneys collected at 0 hr (shame), 6 hr, 24 hr, 72 hr, and 120 hr after ischemic injury are shown for cortex (c) and medulla (m), respectively. The expression levels are shown for embryonic rat kidney (embryonic day 17; E17d) along with those of mouse kidney (E16d and P60d as embryonic day 16 and postnatal day 60, respectively). All data were normalized using GAPDH as a control gene. Immune fluorescence analysis shows the absence of Aurkb expression in sham rat kidney; however, embryonic kidneys and those after 24 hr of ischemic injury show increased Aurkb expression, the nuclear co-localization of which were confirmed by DAPI staining (right). **B**, **C**, **D** are similarly shown for Plk1, Mcm6 and Kif22. 40× magnification.

We selected 4 candidate genes (Aurkb, Plk1, Mcm6, and Kif22) for experimental verification (Figure 6). We confirmed the transcriptional up-regulation of Aurkb, Plk1, Mcm6, and Kif22 after 24 hr of ischemic injury (both in renal cortex and medulla) and also in rat embryonic kidneys (E17d; *n* = 6, each). We also isolated total RNA from 6 day embryo (E16d) and 60-day-old (P60d) BALB/c mice (*n* = 4, each) to confirm the relative up-regulation of these genes in embryonic kidneys. In order to localize these proteins post I/R injury and during development, we performed immunofluorescence analysis using specific antibodies for each protein. While Aurkb and Plk1 staining was absent in sham rats, Mcm6 was localized in the nucleus. At 24 hr post I/R, we observed a marked nuclear staining in the kidney for Aurkb, Plk1 and Mcm6. This effect was also observed in the E17d rat kidneys. The mRNA level of Kif22 also showed significant increased expression at 24 hr after I/R injury while clear decrease was observed after 72 hr and 120 hr. Immunofluorescence analysis shows that Kif proteins are normally located in the nucleus, as confirmed by DAPI nuclear staining. After I/R injury, we observed the increase in Kif staining in the cortico-medullary area of the kidney, where primary ischemic injury occurs. A slight increase in cytoplasmic staining post ischemia for Kif22 was also noted.

## Discussion

Our analysis provides a comprehensive understanding of the functional overlap between the transcriptional programs governing the regenerative and developing kidney processes. These biological processes are regulated by a cascading activation of various molecular functions, which can be inferred from the coordinated expression of functionally related genes [e.g., Gene ontology (GO) annotations]. Among the observed functions, the cell cycle functions were commonly up-regulated during peak of injury (24 hr after I/R injury) and embryonic kidneys (compared to postnatal kidneys) and we validated the transcriptional up-regulation of cell cycle-associated kinases and kinetochore genes, *Aurkb, Plk1, Mcm6, and Kif22*.

Aurkb, Plk1 and Cdc2A are among the chief kinases involving kinetochore assembly and formation of kinetochore-microtubue attachment [16]. The inhibition of Aurkb is associated with a failure in cytokinesis and complete segregation of chromosomes, which leads to polyploidy and cell death in cell culture [17]. *Tpx2* encodes cofactor of Aurora kinases, which is required for correct spindle assembly [18]. *Plk1* also encodes kinase that is essential for cell division [19] and the phosphorylation of this kinase or its substrates by Cdc2A helps the interactions between them [20]. In addition, *Kif22* encodes a chromokinesin involved in chromosome arm orientation on the spindle [21] and similar roles have been reported for Cdca3, Mcm6 and Ranbp1 [22]–[24]. Some studies have proposed the putative roles of kinetochore genes involved in both renal regeneration and development [25]. Our results provide supporting evidences for cell cycle/mitosis-associated molecules as potential markers in evaluating the developing and regenerating kidney. These findings are consistent with the need to proliferate while maintaining fully differentiated function that is shared by the developing and regenerating kidney. Furthermore, genes involved in mitosis checkpoints such as Aurkb and Plk1, are likely to be related to the need for increased surveillance system required in both renal regeneration process and early kidney development.

Prior studies have largely focused on conducting gene expression analysis focusing on one model at-a-time using either regenerating kidney following I/R injury alone or early development of kidney alone and hence have not provided a context to compare two biological processes in terms of expression or functional similarity [4]–[7]. For example, Perco et al observed that the 89 proteins reported in previous I/R injury studies largely represent six functional categories of immunity, cell cycle, apoptosis, cell structure, proliferation, and transporter/carrier [26]. Stuart et al performed expression analysis during the development of rat kidneys [27]. They classified genes associated with different developmental stages into five functional categories, e.g., DNA replication/cell cycle (early embryonic kidney) and transporter/metabolism/oxidative stress functions (adult kidney). Our results are largely consistent with these previous reports; however, we focused on the similarity between I/R injury and developmental kidney expression profiles in terms of enriched GO categories and overlapping genes between the profiles. For example, some of the enriched molecular functions are consistently activated (e.g., cell cycle/apoptosis, immunity/inflammation) or repressed (e.g., membrane-associated enzymes or transporters) during the kidney regeneration or in embryonic kidneys. The similarity of functional annotations between two processes suggests that kidney regeneration after ischemic injury may accompany the reactivation of developmental regulatory programs.

We also observed that hierarchical clustering largely segregates the expression profiles of renal cortex and medulla and the pathway analysis also revealed that some molecular functions are restricted to specific time points or anatomical segments (e.g., the up-regulation of angiogenesis (cortex) and hemopoiesis genes (medulla) in late regenerative phase). The hierarchical clustering of merged dataset also showed that regenerative cortex and medulla resemble the postnatal kidneys of 1 and 7 days, respectively.These findings suggest that these renal segments may have different compensatory mechanism to cope with I/R injury, which are worthy of further investigation. In addition, our analysis involves cross-species comparison (i.e., I/R injury- and development-associated profiles were obtained from rat and mouse, respectively). The expression profiles obtained from rat and mouse would form separate clusters if there were potential batch effects or substantial gene expression differences [28]. However, the hierarchical clustering of merged expression profiles shows that the expression patterns of sham (rat) and postnatal kidneys (PN7d; mouse) are more similar with each other than the remaining expression profiles from the corresponding species. This suggests that the batch effects or the cross-species differences are not substantial compared to I/R injury- or development-associated expression changes.

In summary, the candidate genes identified in this analysis could potentially act as differentiation/dedifferentiation markers and the elucidation of functional significance of their up-regulation in kidney injury and development could offer new therapeutic strategies to enhance kidney regeneration.

## Materials and Methods

### Animals

Male Wistar rats (280–300 g, 8–9 weeks old) were purchased from Harlan Laboratories (Indianapolis, IN) andwere maintained in central animal facility over wood chips free of any known chemical contaminants under conditions of 21±1°C and 50–80% relative humidity at all times in an alternating 12 hr light-dark cycle. Animals were fed with commercial rodent chow (Teklad rodent diet # 7012), given water ad lib, and were acclimated for 1-week prior to use. All animal maintenance and treatment protocols were in compliance with the Guide for Care and Use of Laboratory animals as adopted and promulgated by the National Institutes of Health and were approved by the Harvard Medical School Animal Care and Use Committees (IACUC).

### Experimental design

Nine male Wistar rats underwent I/R surgery and three rats underwent sham surgery simulating I/R. In order to perform I/R surgery, the rats were anesthetized using pentobarbital sodium (30 mg/kg, ip) and renal ischemia was induced by nontraumatic vascular clamps over the pedicles for 20 min as described before [3], [29]. Upon release of the clamps, the incision was closed in two layers with 2–0 sutures. The sham rats underwent anesthesia and a laparotomy only and were sacrificed after 24 hr. The rats in I/R group were further divided in subgroups of three rats each and sacrificed after 6 hr, 24 hr, and 120 hr of reperfusion. To confirm the results of gene expression analysis twenty male Wistar rats underwent 20 minutes bilateral I/R surgery and five rats underwent sham surgery as described above and were sacrificed at 6 hr, 24 hr, 72 hr and 120 hr following reperfusion (*n* = 5, each timepoint). At the time of sacrifice, kidneys were macroscopically divided into renal cortex and medulla, frozen in liquid nitrogen, and maintained at −80°C until use as previously described [30]. We also included rat embryos at embryonic day 17 p.c. for kidney development analysis (E17d). Kidney was collected from male BALBc mice embryos at embryonic day 16 p.c. (E16d) as well as those from 1, 7 and 60 postnatal days (PN1d, PN7d and P60d, respectively). The E17d rat embryonic kidneys were collected from Sprague-Dawleyrats from Charles River Laboratories, Inc. (Raleigh, NC).

### Blood Chemistry

At sacrifice of rats following renal I/R injury, blood was collected from dorsal aorta in heparinized tubes. Serum creatinine (SCr) concentrations were measured using a Beckman Creatinine Analyzer II. Blood urea nitrogen (BUN) was measured spectrophotometrically at 340 nm using a commercially available kit (Thermo Scientific, Rockford, IL) as described before [3].

### RNA isolation and Real Time-Polymerase Chain Reaction (RT-PCR)

Total RNA was isolated from each kidney cortex, medulla, and embryonic kidney following the manufacturer's protocol for Trizol extraction method (Invitrogen, Carisbad, CA). The density of total RNA was measured by spectrophotometer, and subsequently checked for integrity by 1% agarose gel electrophoresis. All RNA samples were treated with DNase I (Invitrogen, Carisbad; CA) to avoid DNA contamination followed by reverse transcription with 1 µg of total RNA using iScriptcDNA synthesis (BIO-RAD. Hercules; CA). The mRNA levels of Kim-1, Vimentin, Kif22, Mcm6, Aurkb, Plk1 and GAPDH as the internal control were determined using the iQ SYBR Green super mix kit (Bio-rad, Hercules, CA) following the manufacturer's instructions. We performed the assay in an iCycler PCR System (BIO-RAD, Hercules, CA) with the cycling protocol as follows: 3′94°C, 15″55°C, and 72°C for 35″ for 45 cycles. The primer pair sequence of each assay is detailed in Table 1. The relative quantification of gene expression was performed using the comparative CT method.

**Table 1:** The primer pairs for RT-PCR.

Gene	Forward primer	Reverse primer
Kim-1 (rat)	5′ CCACAACTACAAGACCCACAACCAC 3′	5′ GGATGTCACAGTGCCATTCCAG 3′
Kim-1 (mouse)	5′ GGAAGTAAAGGGGGTAGTGGG 3′	5′ AAGCAGAAGATGGGCATTGC 3′
Vimentin	5′ ACCGCTTCGCCAACTACATC 3′	5′ GCAACTCCCTCATCTCCTCC 3′
Kif22	5′ CTAAGCAAGGGAGGAGTCAGC 3′	5′ TTGGCTACTTCAAGAGAGCAGC 3′
Mcm6	5′ CCGAATCTCTAACCTCATCGTGC 3′	5′ GCTCTCGCTTCCTTCACTGGAG 3′
Aurkb	5′ GATGATTGAAGGGCGGATGC 3′	5′ AAAGGGCAGAGGGAGGCAGAAC 3′
Plk1 (rat)	5′ TGCTCAAGCCCCATCAGAAG 3′	5′ AGTCGCTGTCCTCAAAAAAGC 3′
Plk1 (mouse)	5′ CCCCACCAGAAGGAGAAGATG 3′	5′ AAATACAAATACAAAGTCGCTGTCC 3′
GAPDH	5′ TCCGCCCCTTCTGCCGATG 3′	5′ CACGGAAGGCCATGCCAGTGA 3′

### Separation of cortex and medulla

To confirm that there was no contamination between cortex and medulla, the mRNA levels for sodium chloride cotransporter (NCC) as a specific gene expressed in cortex was quantified by real-time PCR on the ABI Prism 7300 Sequence Detection System (*Taq*Man, ABI, Foster City, CA, assay number assay number Rn00571074_m1). As an endogenous control, we used eukaryotic 18S rRNA (predesigned assay reagent, external run, ABI). The relative quantification of expression was performed using the comparative CT method [31] Results revealed that mRNA levels of NCC were abundantly expressed in renal cortex (1±0.07) as compared to renal medulla (0.001±0.0001) demonstrating that there was no contamination of the cortex with medulla (Figure S1).

### Microarray analysis

Biotin-16-UTP was used during in vitro transcription. Microarray analysis was performed in biological triplicates. Labeled RNAs were hybridized onto Gene chip array RatRef-12 V1.0 (BD-202-0202 Illumina San Diego CA.), which contains 22,523 genes for rat. For mouse RNA, we used MouseRef-8 V1.1 (AMIL1791 Illumina San Diego; CA.) with 24,854 genes following the manufacturer's instructions. After washing and blocking, streptavidin-Cy3 was used to crosslink the amplified RNA, which were scanned by the Illumina bead reader. The data was analyzed using Illumina bead studio software (Illumina San Diego; CA). The scanned intensity values were quantile normalized for subsequent analysis. The expression microarray data can be accessed from Gene Expression Omnibus (GEO) with accession number of GSE27274 (rat) and GSE28054 (mouse).

### Pathway analysis

Agglomerative hierarchical clustering was performed for the highly variable 2000 genes from expression profiles ofrenal I/R injury (rat) and nephrogenic models (mouse). For clustering, 1 minus Pearson correlation coefficient was used as distance with average linkage [32]. For functional gene set, we obtained GO categories from MSigDB database (http://www.broadinstitute.org/gsea/msigdb/index.jsp; c5 GO category) [33]. Pathway analysis was performed using parametric analysis of gene expression (PAGE) method [14]. For individual GO categories, the significance of enrichment was measured by converting the mean fold change of genes belonging to the GO category into *Z* score. The significance of enrichment was measured across 6 comparisons (3 time points after I/R injury against sham, cortex and medulla, respectively) and 2 comparisons (PN1d and PN7d against E16d). Significantly (Bonferroni corrected *P*<0.05) enriched GO categories were collected with leading edge gene subsets (fold change >1.5 or <−1.5). A full list of enriched GO categories are available in Tables S1,S2,S3 (I/R injury in cortex and medulla as well as mouse developmental kidney). To construct functional network of gene sets or association map, we first collected genes showing highly up- or down-regulation (fold change >1.5 or <−1.5, respectively) for 8 time scales (6 for rat kidney and 2 for mouse kidney) [34]. Sixteen sets of differentially expressed genes were then measured for the significant of enrichment with GO categories by Fisher's exact test and 57 GO categories showing significant enrichment (Bonferroni corrected *P*<0.05) with any of 16 gene sets were selected. We ignored the down-regulated genes in PN1d in network since they do not show significant association with other gene sets. The selected GO categories were manually curated into 6 functional categories (cell cycle/mitosis, immune/inflammation, transport, metabolism, cytoskeleton, and others). The collected 72 gene sets (57 GO categories and 15 regeneration-/development-associated gene sets) were measured for the significance of enrichment in a pairwise manner. We used CytoScape software to construct a network using organic layout [35]. In the network, the gene sets are represented as nodes and the significant association between the gene sets was shown as edge.

### Immunofluorescence

After perfusion, the kidneys were isolated, frozen and later sectioned in slides of 5 µm thickness. For single staining, cryo-sections from 4 rats of each group and three E17d tissues were fixed in 10% formaldehyde for 5 min, followed by 5 min 0.1% Triton-X permeabilization, and then blocking with BSA 3% for 60 min. After washing, the sections were incubated overnight with rabbit monoclonal anti-Ki67 (Vector Laboratories, Burlingame, CA), rabbit anti Kif22 1∶1000 (Abcam, Cambridge MA), rabbit anti Plk1 antibody 1∶500 (Cell Signaling Technology Inc), and rabbit anti Aurkb (1∶500) (Cell Signaling Technology Inc, Danvers.MA). Goat anti-mcm6 antibody 1∶1000 (Abcam, Cambridge MA.) at 4°C was used. After washing, sections were incubated at room temperature with secondary antibody Cy3 conjugated goat anti rabbit 1∶600 (Jackson Immune research, West Grove Pa) or alexafluor 488 conjugated donkey anti goat 1∶500 (Invitrogen) for 60 min and washed again. The images were captured by Nikon DS-QiMc camera attached to Nikon eclipse 90i flourescence microscope using oil immersion objectives (40× magnification) by Nikon NIS elements AR ver3.2 software.

### Statistics

Data are expressed as average+standard error. Statistical difference (*P*<0.05) as calculated by one way ANOVA or student's t-test. *P*<0.05 was considered significant and represented by ‘*’ where applicable. All graphs were generated by GraphPad Prism (GraphPad, Inc., La Jolla, CA).

## Supporting Information

Figure S1 **Realtime PCR analysis confirming no cross contamination between kidney cortex and medulla.** Real time-PCR for sodium chloride cotransporter (NCC) that is expressed only in the cortex was conducted. Results were normalized to eukaryotic 18S rRNA as control gene. * represents *p*<0.05 determined by t test with respect to renal cortex.
(TIF)

Table S1 **The GO functional categories significantly (Bonferroni corrected P<0.05) enriched in cortex I/R injury profiles.** The functions up- and down-regulated in each time scale (6 hr, 24 hr and 120 hr) compared to Sham are shown. The gene size and uncorrected significance level are also shown. Leading edge genes represent genes highly up- and down-regulated (fold change >1.5 and <−1.5) among the genes belonging to the corresponding GO categories.
(PDF)

Table S2 **The GO functional categories significantly enriched in medulla I/R injury profiles.**
(PDF)

Table S3 **The GO functional categories significantly enriched in developing kidney.** The expression profiles from postnatal day 1 and 7 (PN1d and PN7d) are compared to that of embryonic kidney (E16d).
(PDF)
