# Supplementary figures and images for: Gene Expression Analysis Reveals the Cell Cycle and Kinetochore Genes Participating in Ischemia Reperfusion Injury and Early Development in Kidney
Source: PLoS One. 2011 Sep 28;6(9):e25679. doi: 10.1371/journal.pone.0025679 (PMC3181346; doi:10.1371/journal.pone.0025679)

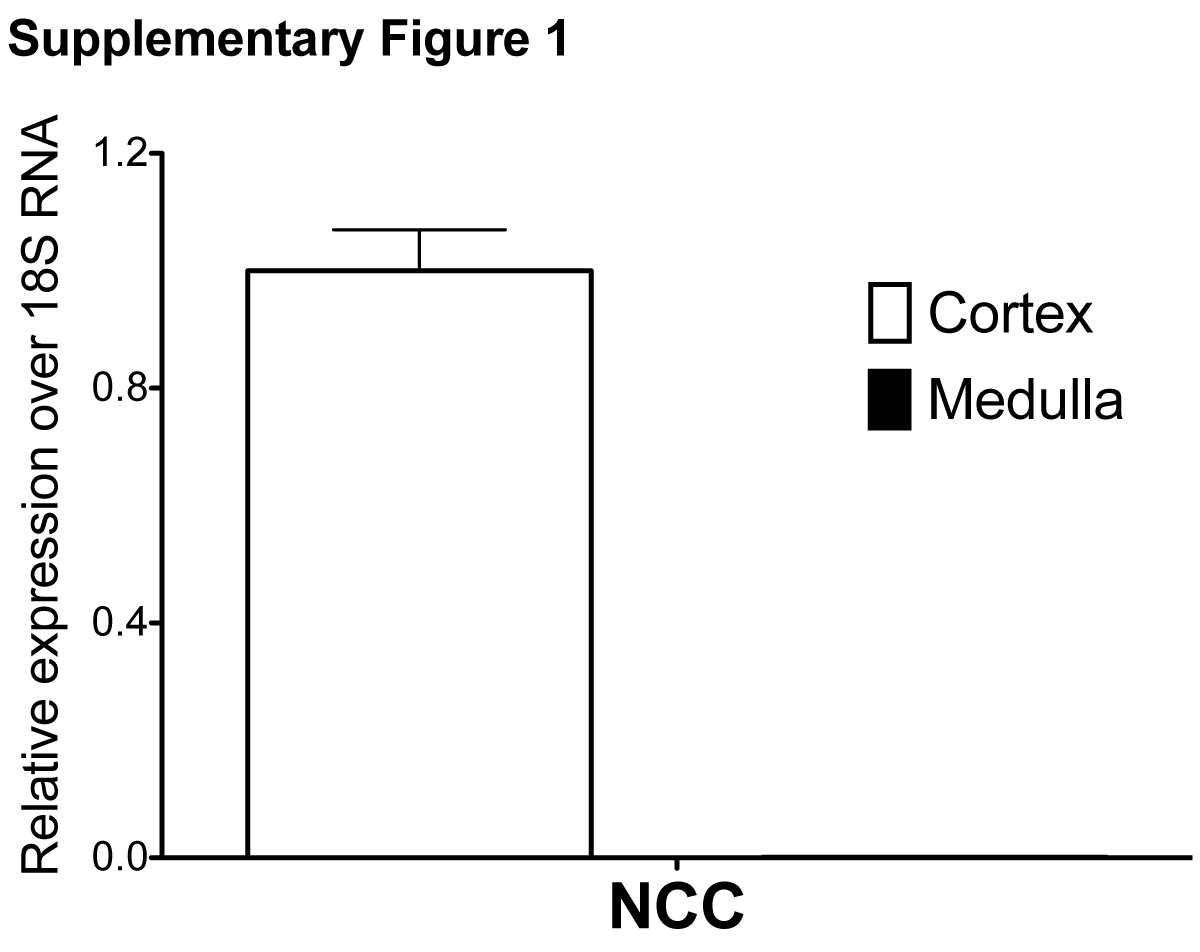

Supplement: Figure S1 — Realtime PCR analysis confirming no cross contamination between kidney cortex and medulla. Real time-PCR for sodium chloride cotransporter (NCC) that is expressed only in the cortex was conducted. Results were normalized to eukaryotic 18S rRNA as control gene. * represents p<0.05 determined by t test with respect to renal cortex. (TIF) [file pone.0025679.s001.tif]
