# Supplementary material for: Gene Expression Analysis Reveals the Cell Cycle and Kinetochore Genes Participating in Ischemia Reperfusion Injury and Early Development in Kidney
Source: PLoS One. 2011 Sep 28;6(9):e25679. doi: 10.1371/journal.pone.0025679 (PMC3181346; doi:10.1371/journal.pone.0025679)
Supplement: Table S1 — The GO functional categories significantly (Bonferroni corrected P<0.05) enriched in cortex I/R injury profiles. The functions up- and down-regulated in each time scale (6 hr, 24 hr and 120 hr) compared to Sham are shown. The gene size and uncorrected significance level are also shown. Leading edge genes represent genes highly up- and down-regulated (fold change >1.5 and <−1.5) among the genes belonging to the corresponding GO categories. (PDF) [file pone.0025679.s002.pdf]

Supplementary Table 1. The GO functional categories significantly (Bonferroni corrected P < 0.05) enriched in cortex I/R injury profiles. The functions up- and down-regulated in each time scale (6hr, 24hr and 120hr) compared to Sham are shown. The gene size and significance level (uncorrected) are also shown. Leading edge genes represent genes highly up- and down-regulated (fold change > 1.5 and < -1.5) among the genes beloning to the corresponding GO categories.

Scale	GO category	Gene	Pvalue	Leading edge genes
Up (6hr)	PROTEASOME COMPLEX	18	7.647E-13	
	RESPONSE_TO_STRESS	351	6.149E-11	CCL20,CXCL10,HSPB1,CLDN3,S100A8,GADD45G,BTG2
	DEFENSE RESPONSE	175	1.809E-09	S100A8,CCL20,CXCL10,FOSL1
	INFLAMMATORY RESPONSE	96	7.742E-09	S100A8,CXCL10,CCL20
	CHEMOKINE RECEPTOR BINDING	26	2.708E-08	CXCL10,CCL20
	G PROTEIN COUPLED RECEPTOR BINDING	33	2.832E-08	CXCL10,CCL20
	CHEMOKINE ACTIVITY	25	3.05E-08	CXCL10,CCL20
	REGULATION_OF_CELL_PROLIFERATION	227	5.095E-08	RARRES1,ADAMTS1,CXCL10,EMP3,TIMP1,FOSL1,BTG2
	NEGATIVE_REGULATION_OF_BIOLOGICAL_PROCESS	457	5.642E-08	CRYAB,ADAMTS1,HSPB1,TIMP1,RARRES1,EMP3,BTG2
	CYTOKINE ACTIVITY	68	1.105E-07	CXCL10,CCL20
	CELL DEVELOPMENT	421	1.724E-07	CRYAB,HSPB1
	NEGATIVE_REGULATION_OF_CELLULAR_PROCESS	437	2.111E-07	CRYAB,ADAMTS1,HSPB1,TIMP1,RARRES1,EMP3,BTG2
	NEGATIVE REGULATION OF DEVELOPMENTAL PROCESS	146	3.342E-07	CRYAB,HSPB1
	PROGRAMMED CELL DEATH	315	3.364E-07	CRYAB,HSPB1
	APOPTOSIS GO	314	4.288E-07	CRYAB,HSPB1
	NEGATIVE REGULATION OF PROGRAMMED CELL DEATH	113	4.581E-07	CRYAB,HSPB1
	NEGATIVE REGULATION OF APOPTOSIS	112	6.534E-07	CRYAB,HSPB1
	CELL_PROLIFERATION_GO_0008283	364	9.197E-07	ADAMTS1,CXCL10,TIMP1,FOSL1,RARRES1,EMP3,BTG2
	REGULATION OF DEVELOPMENTAL PROCESS	312	9.484E-07	CRYAB,HSPB1
	REGULATION OF I KAPPAB KINASE NF KAPPAB CASCADE	60	9.636E-07	HMOX1
	MACROMOLECULAR COMPLEX	598	2.046E-06	
	POSITIVE_REGULATION_OF_I_KAPPAB_KINASE_NF_KAPPAB_CASCADE	58	2.54E-06	HMOX1
	POSITIVE REGULATION OF BIOLOGICAL PROCESS	461	2.803E-06	CXCL10,TIMP1,HMOX1,FOSL1
	EXTRACELLULAR SPACE	173	2.873E-06	PVR,CCL20,IGFBP1
	TRANSLATION	119	2.95E-06	HSPB1
	POSITIVE REGULATION OF CELLULAR PROCESS	436	4.14E-06	CXCL10,TIMP1,HMOX1,FOSL1
	REGULATION OF PROGRAMMED CELL DEATH	246	4.317E-06	CRYAB,HSPB1
	CELL CELL SIGNALING	302	4.697E-06	CCL20,CXCL10,NQO1
	POSITIVE REGULATION OF SIGNAL TRANSDUCTION	80	4.812E-06	HMOX1
	ANTI APOPTOSIS	86	5.138E-06	CRYAB,HSPB1
	REGULATION OF APOPTOSIS	245	5.534E-06	CRYAB,HSPB1
	LOCOMOTORY BEHAVIOR	70	5.816E-06	CXCL10,FOSL1,CCL20
	INTRACELLULAR SIGNALING CASCADE	452	7.494E-06	HMOX1,GADD45G
	POSITIVE REGULATION OF CELL PROLIFERATION	110	1.032E-05	CXCL10,TIMP1,FOSL1
	EXTRACELLULAR REGION	320	1.19E-05	CCL20,PVR,IGFBP1
	RRNA METABOLIC PROCESS	10	1.572E-05	
	EXTRACELLULAR REGION PART	238	1.746E-05	PVR,CCL20,IGFBP1
	CELLULAR BIOSYNTHETIC PROCESS	214	1.912E-05	HSD3B1,NQO1,HSPB1
	IMMUNE SYSTEM PROCESS	228	1.931E-05	CCL20
	PROTEIN COMPLEX	526	2.354E-05	
	CALCIUM INDEPENDENT CELL CELL ADHESION	16	3.167E-05	CLDN3
	DNA METABOLIC PROCESS	170	3.829E-05	GADD45G,BTG2
Down (6hr)	GOLGI_APPARATUS_PART	66	1.527E-06	TGOLN2,STEAP2
	GOLGI MEMBRANE	29	2.956E-06	STEAP2
	MITOCHONDRION	217	1.362E-05	GATM,MAOB
Up (24hr)	MITOTIC_CELL_CYCLE	106	9.949E-19	PRC1,KNTC1,TTK,CCNA2,PLK1,KIF23,KIF22,DLG7,TPX2,UBE2C,BUB1,CDKN3
	NON_MEMBRANE_BOUND_ORGANELLE	359	4.148E-15	TPX2,BUB1,CCNB2,PRC1,KNTC1,ARPC1B,HMGB2,PLK1,KIF23,KIF22,DLG7,CDC20,TTK,NOLA2
	INTRACELLULAR_NON_MEMBRANE_BOUND_ORGANELLE	359	4.148E-15	TPX2,BUB1,CCNB2,PRC1,KNTC1,ARPC1B,HMGB2,PLK1,KIF23,KIF22,DLG7,CDC20,TTK,NOLA2
	CELL_CYCLE_PROCESS	134	8.414E-15	CCNA2,TPX2,BUB1,CDKN3,PRC1,KNTC1,TTK,PLK1,KIF23,KIF22,DLG7,UBE2C
	STRUCTURAL MOLECULE ACTIVITY	162	8.642E-15	FBLN1,MGP,ARPC1B
	STRUCTURAL CONSTITUENT OF RIBOSOME	72	1.423E-14	
	CHROMOSOMAL PART	60	1.56E-14	KIF22,BUB1
	CELL_CYCLE_GO_0007049	210	2.722E-14	CCNA2,CDC20,TPX2,BUB1,GMNN,CDKN3,PRC1,KNTC1,TTK,PLK1,KIF23,KIF22,DLG7,UBE2C
	M_PHASE_OF_MITOTIC_CELL_CYCLE	59	1.537E-13	KIF22,KNTC1,TTK,DLG7,CCNA2,TPX2,UBE2C,BUB1,PLK1
	M_PHASE	80	4.539E-13	KNTC1,TTK,CCNA2,PLK1,KIF22,DLG7,TPX2,UBE2C,BUB1
	CHROMOSOME	77	1.292E-12	HMGB2,KIF22,BUB1
	SPINDLE	30	1.43E-12	KIF23,PRC1,KNTC1,TTK,DLG7,BUB1,TPX2,CDC20
	MITOSIS	57	4.171E-12	KIF22,KNTC1,TTK,CCNA2,TPX2,UBE2C,BUB1,PLK1
	CELL_CYCLE_PHASE	119	1.57E-11	KNTC1,TTK,CCNA2,PLK1,KIF22,DLG7,TPX2,UBE2C,BUB1,CDKN3
	REPLICATION FORK	13	2.84E-10	

	EXTRACELLULAR_REGION_PART	238	4.65E-10	PVR,WFDC2,CCL20,FBLN1,MGP,FGG,FGB,HPX,IGFBP1
	DNA METABOLIC PROCESS	170	1.742E-09	S100A11,GMNN,HMGB2
	TRANSLATION	119	3.999E-09	
	RESPONSE TO DNA DAMAGE STIMULUS	103	6.037E-09	CCNA2,HMGB2
	EXTRACELLULAR_REGION	320	6.942E-09	WFDC2,CCL20,MGP,FGG,FGB,PVR,FBLN1,HPX,IGFBP1
	EXTRACELLULAR_SPACE	173	8.95E-09	PVR,WFDC2,CCL20,FBLN1,FGG,FGB,HPX,IGFBP1
	DNA REPLICATION	67	1.264E-08	HMGB2,S100A11,GMNN
	RESPONSE_TO_STRESS	351	1.394E-08	CCNA2,CCL20,CXCL11,CXCL10,HMGB2,CLDN3
	MITOTIC CELL CYCLE CHECKPOINT	16	3.712E-08	KNTC1,TTK,BUB1,CCNA2
	CHROMOSOME PERICENTRIC REGION	17	5.133E-08	KIF22,BUB1
	MACROMOLECULAR_COMPLEX	598	5.872E-08	KIF23,KIF22,BUB1,ARPC1B,FGG,FGB,SNRPA,NOLA2
	NUCLEUS	836	6.632E-08	PRC1,KIF22,CCNA2,TPX2,HMGB2,DLG7,S100A6,LGALS3,S100A11,NUPR1,SNRPA,NOLA2,NRM
	KINETOCHORE	16	1.101E-07	KIF22,BUB1
	CHEMOKINE ACTIVITY	25	1.669E-07	CXCL11,CXCL10,CCL20
	CHEMOKINE RECEPTOR BINDING	26	3.337E-07	CXCL11,CXCL10,CCL20
	CELL_PROLIFERATION_GO_0008283	364	3.443E-07	ADAMTS1,TPX2,BUB1,CXCL10,TIMP1,PLK1,S100A11,CDKN3,TTK,EMP3
	DEFENSE RESPONSE	175	3.546E-07	CCL20,CXCL11,CXCL10
	DNA DEPENDENT DNA REPLICATION	37	6.174E-07	HMGB2,GMNN,S100A11
	RESPONSE TO ENDOGENOUS STIMULUS	129	6.337E-07	CCNA2,HMGB2
	REGULATION OF MITOSIS	31	8.814E-07	KNTC1,TTK,BUB1,CCNA2,UBE2C
	MICROTUBULE_CYTOSKELETON	93	9.703E-07	PRC1,KNTC1,TTK,CDC20,PLK1,KIF23,DLG7,TPX2,BUB1,CCNB2
	INFLAMMATORY RESPONSE	96	1.021E-06	CXCL11,CXCL10,CCL20
	REGULATION OF DEVELOPMENTAL PROCESS	312	1.052E-06	CRYAB
	DNA REPAIR	82	1.94E-06	HMGB2
	PROTEIN COMPLEX	526	2.097E-06	KIF23,KIF22,BUB1,ARPC1B,FGG,FGB
	CELLULAR BIOSYNTHETIC PROCESS	214	2.311E-06	
	CYTOSKELETAL_PART	132	2.717E-06	CDC20,TPX2,BUB1,PRC1,KNTC1,TTK,ARPC1B,PLK1,KIF23,DLG7
	G PROTEIN COUPLED RECEPTOR BINDING	33	2.916E-06	CXCL11,CXCL10,CCL20
	PROGRAMMED CELL DEATH	315	3.056E-06	CRYAB
	SPINDLE POLE	16	3.177E-06	KNTC1,TPX2,DLG7,BUB1
	APOPTOSIS GO	314	3.47E-06	CRYAB
	REGULATION OF PROGRAMMED CELL DEATH	246	4.517E-06	CRYAB
	PROTEASOME COMPLEX	18	5.096E-06	
	REGULATION OF APOPTOSIS	245	5.181E-06	CRYAB
	NUCLEAR CHROMOSOME	32	7.883E-06	HMGB2
	CELL DEVELOPMENT	421	8.398E-06	CRYAB
	REGULATION_OF_CELL_PROLIFERATION	227	1.658E-05	ADAMTS1,S100A11,CDKN3,TTK,CXCL10,EMP3,TIMP1
	RRNA METABOLIC PROCESS	10	1.871E-05	NOLA2
	CYTOKINE ACTIVITY	68	1.926E-05	IL19,CXCL11,CXCL10,CCL20
	NEGATIVE REGULATION OF DEVELOPMENTAL PROCESS	146	2.209E-05	CRYAB
	CYTOSKELETON	210	2.557E-05	CDC20,TPX2,BUB1,CCNB2,PRC1,KNTC1,TTK,ARPC1B,PLK1,KIF23,DLG7
	NEGATIVE REGULATION OF PROGRAMMED CELL DEATH	113	2.928E-05	CRYAB
	NEGATIVE REGULATION OF APOPTOSIS	112	3.454E-05	CRYAB
	MACROMOLECULE BIOSYNTHETIC PROCESS	205	3.476E-05	
	NUCLEAR PART	318	3.939E-05	HMGB2,SNRPA,NOLA2,NRM
Down (24hr)	MITOCHONDRION	217	1.012E-10	PDHA1,GATM,TBRG4,DMGDH
	ORGANIC ACID METABOLIC PROCESS	130	2.44E-07	GATM
	CARBOXYLIC ACID METABOLIC PROCESS	128	6.533E-07	GATM
	GOLGI APPARATUS PART	66	3.994E-06	TGOLN2,STEAP2
	OXIDOREDUCTASE_ACTIVITY__ACTING_ON_THE_ALDEHYDE_OR_OXO_GROUP OF DONORS	15	6.216E-06	ALDH8A1,ALDH9A1
	SECONDARY_ACTIVE_TRANSMEMBRANE_TRANSPORTER_ACTIVITY	35	8.538E-06	SLC5A2
	SYMPORTER ACTIVITY	26	2.699E-05	SLC5A2
	AMINO ACID AND DERIVATIVE METABOLIC PROCESS	80	3.19E-05	GATM
Up (120hr)	STRUCTURAL_CONSTITUENT_OF_RIBOSOME	72	2.04E-26	
	STRUCTURAL MOLECULE ACTIVITY	162	3.543E-26	
	CHEMOKINE ACTIVITY	25	1.638E-22	CXCL1,CCL2,CXCL11,CXCL10,CCL20
	CHEMOKINE RECEPTOR BINDING	26	5.731E-22	CXCL1,CCL2,CXCL11,CXCL10,CCL20
	EXTRACELLULAR REGION PART	238	7.257E-22	PVR,CXCL1,CCL20,C2,IL1B,CCL2,FGB,IGFBP1
	G PROTEIN COUPLED RECEPTOR BINDING	33	1.103E-19	CXCL11,CXCL10,CXCL1,CCL2,CCL20
	EXTRACELLULAR REGION	320	7.77E-19	CCL20,C2,IL1B,CCL2,FGB,PVR,CXCL1,IGFBP1
	DEFENSE RESPONSE	175	1.915E-18	CXCL1,CCL20,C2,CXCL11,CXCL10
	INFLAMMATORY RESPONSE	96	3.239E-18	CXCL11,CXCL10,CXCL1,CCL20,C2
	IMMUNE SYSTEM PROCESS	228	1.674E-16	CCL20,C2,CCL2
	CYTOKINE ACTIVITY	68	1.96E-15	IL19,CXCL11,CXCL10,CCL2,CXCL1,CCL20
	EXTRACELLULAR SPACE	173	1.395E-13	PVR,CXCL1,CCL20,C2,IL1B,CCL2,FGB,IGFBP1
	IMMUNE RESPONSE	161	1.78E-13	CCL20,C2,CCL2
	NEGATIVE_REGULATION_OF_BIOLOGICAL_PROCESS	457	4.929E-13	ADAMTS1,HPGD,IL1B,CCL2,TIMP1,S100A11,CXCL1

	LOCOMOTORY BEHAVIOR	70	1.84E-12	CXCL11,CXCL10,CCL2,CXCL1,CCL20
	REGULATION OF DEVELOPMENTAL PROCESS	312	3.32E-12	CCL2
	RESPONSE TO STRESS	351	4.78E-12	CCL20,C2,CXCL11,CXCL10,CLDN3,CXCL1
	NEGATIVE_REGULATION_OF_CELLULAR_PROCESS	437	4.861E-12	ADAMTS1,HPGD,IL1B,CCL2,TIMP1,S100A11,CXCL1
	RESPONSE TO WOUNDING	142	5.091E-12	CXCL11,CXCL10,CXCL1,CCL20,C2
	PROTEINACEOUS EXTRACELLULAR MATRIX	70	6.766E-12	
	EXTRACELLULAR MATRIX	72	1.521E-11	
	PROGRAMMED CELL DEATH	315	7.813E-11	IL1B,CCL2
	APOPTOSIS GO	314	8.361E-11	IL1B,CCL2
	TRANSLATION	119	8.633E-11	
	RESPONSE TO EXTERNAL STIMULUS	228	1.642E-10	CXCL1,CCL20,C2,CXCL11,CXCL10,CCL2
	HUMORAL IMMUNE RESPONSE	20	2.082E-10	CCL2,C2
	COLLAGEN	18	4.514E-10	
	POSITIVE REGULATION OF BIOLOGICAL PROCESS	461	7.413E-10	C2,CXCL10,TIMP1
	REGULATION OF CELL PROLIFERATION	227	9.682E-10	ADAMTS1,S100A11,CXCL1,CXCL10,IL1B,TIMP1
	CELL DEVELOPMENT	421	2.846E-09	IL1B,CCL2
	REGULATION OF PROGRAMMED CELL DEATH	246	4.806E-09	CCL2
	REGULATION OF APOPTOSIS	245	5.145E-09	CCL2
	POSITIVE REGULATION OF CELLULAR PROCESS	436	5.745E-09	CXCL10,TIMP1
	CELL PROLIFERATION GO 0008283	364	1.286E-08	ADAMTS1,CXCL10,IL1B,TIMP1,S100A11,CXCL1
	EXTRACELLULAR MATRIX PART	35	1.84E-08	
	NON MEMBRANE BOUND ORGANELLE	359	3.988E-08	CCNB2,PRC1
	INTRACELLULAR NON MEMBRANE BOUND ORGANELLE	359	3.988E-08	CCNB2,PRC1
	CELLULAR BIOSYNTHETIC PROCESS	214	4.529E-08	HPGD
	RECEPTOR BINDING	246	9.207E-08	CXCL1,CCL20,IL19,CXCL11,CXCL10,CCL2
	NEGATIVE REGULATION OF DEVELOPMENTAL PROCESS	146	1.151E-07	CCL2
	BIOSYNTHETIC PROCESS	303	4.552E-07	HPGD
	MULTICELLULAR ORGANISMAL DEVELOPMENT	711	4.929E-07	CXCL10,CCL2,CXCL1,TNFRSF12A,LAMC2
	REGULATION OF I KAPPAB KINASE NF KAPPAB CASCADE	60	6.156E-07	
	NEGATIVE REGULATION OF PROGRAMMED CELL DEATH	113	6.467E-07	CCL2
	NEGATIVE REGULATION OF APOPTOSIS	112	6.888E-07	CCL2
	PROTEIN METABOLIC PROCESS	809	7.464E-07	C2,CCL2,TIMP1
	ANATOMICAL STRUCTURE DEVELOPMENT	688	1.083E-06	CXCL10,CCL2,CXCL1,LAMC2
	CELLULAR PROTEIN METABOLIC PROCESS	740	1.524E-06	C2,CCL2,TIMP1
	PROTEOLYSIS	129	1.733E-06	TIMP1,C2
	SYSTEM DEVELOPMENT	595	2.262E-06	CXCL10,CCL2,CXCL1,LAMC2
	POSITIVE_REGULATION_OF_I_KAPPAB_KINASE_NF_KAPPAB_CASCADE	58	2.264E-06	
	CELLULAR MACROMOLECULE METABOLIC PROCESS	749	2.267E-06	C2,CCL2,TIMP1
	CELLULAR DEFENSE RESPONSE	35	2.453E-06	
	EPIDERMIS DEVELOPMENT	40	3.216E-06	LAMC2
	INTRACELLULAR SIGNALING CASCADE	452	3.301E-06	CCL2,CXCL1
	BEHAVIOR	118	3.474E-06	CXCL11,CXCL10,CCL2,CXCL1,CCL20
	MACROMOLECULE BIOSYNTHETIC PROCESS	205	3.583E-06	
	NEGATIVE REGULATION OF CELL PROLIFERATION	117	3.733E-06	IL1B,ADAMTS1,S100A11,CXCL1
	MITOTIC CELL CYCLE	106	3.816E-06	PRC1
	MACROMOLECULAR COMPLEX	598	4.356E-06	FGB
	ECTODERM DEVELOPMENT	42	4.682E-06	LAMC2
	POSITIVE REGULATION OF SIGNAL TRANSDUCTION	80	6.003E-06	
	POSITIVE REGULATION OF CELL PROLIFERATION	110	7.308E-06	CXCL10,TIMP1
	RNA BINDING	169	1.232E-05	
	POSITIVE REGULATION OF DEVELOPMENTAL PROCESS	150	1.353E-05	
	PROTEIN KINASE CASCADE	193	1.501E-05	CCL2
	ADAPTIVE IMMUNE RESPONSE	17	1.825E-05	C2
	TISSUE DEVELOPMENT	84	2.206E-05	LAMC2
	ANTI APOPTOSIS	86	2.515E-05	CCL2
	PROTEASOME COMPLEX	18	2.641E-05	
	REGULATION OF ANGIOGENESIS	19	2.969E-05	
	ICOSANOID METABOLIC PROCESS	10	3.747E-05	HPGD
	EXTRACELLULAR MATRIX STRUCTURAL CONSTITUENT	20	4.03E-05	
Down (120hr)	MITOCHONDRION	217	1.085E-07	MAOB
	GOLGI APPARATUS PART	66	3.139E-06	
	ORGANIC ACID METABOLIC PROCESS	130	4.496E-06	
	CARBOXYLIC ACID METABOLIC PROCESS	128	6.055E-06	
	SECONDARY_ACTIVE_TRANSMEMBRANE_TRANSPORTER_ACTIVITY	35	1.52E-05	
	SYMPORTER ACTIVITY	26	1.749E-05	
	AMINO ACID METABOLIC PROCESS	61	2.126E-05	
	CARBOHYDRATE TRANSMEMBRANE TRANSPORTER ACTIVITY	14	2.55E-05	
