# Supplementary material for: Gene Expression Analysis Reveals the Cell Cycle and Kinetochore Genes Participating in Ischemia Reperfusion Injury and Early Development in Kidney
Source: PLoS One. 2011 Sep 28;6(9):e25679. doi: 10.1371/journal.pone.0025679 (PMC3181346; doi:10.1371/journal.pone.0025679)
Supplement: Table S2 — The GO functional categories significantly enriched in medulla I/R injury profiles. (PDF) [file pone.0025679.s003.pdf]

Supplementary Table 2. The GO functional categories significantly enriched in medulla I/R injury profiles.

Scale	GO category	Gene	Pvalue	Leading edge genes
Up (6hr)	REGULATION_OF_CELL_PROLIFERATION	227	5.256E-16	ADAMTS1,SPHK1,S100A11,CXCL1,CXCL10,EMP3,IFITM1,TIMP1,FOSL1,BTG2
	POSITIVE_REGULATION_OF_BIOLOGICAL_PROCESS	461	2.062E-14	SPHK1,CXCL10,TIMP1,HMOX1,FOSL1,FST,LGALS1,EGR1,TNFRSF1A
	POSITIVE_REGULATION_OF_CELLULAR_PROCESS	436	6.82E-14	SPHK1,CXCL10,TIMP1,HMOX1,FOSL1,LGALS1,EGR1,TNFRSF1A
	STRUCTURAL_MOLECULE_ACTIVITY	162	7.162E-14	MATN1,FBLN1,ARPC1B,ARPC3,ANXA1
	CHEMOKINE_ACTIVITY	25	1.361E-13	CXCL1,CXCL11,CXCL10
	DEFENSE_RESPONSE	175	1.427E-13	S100A8,ELF3,CXCL1,CXCL11,CXCL10,ADORA2A,FOSL1,TNFRSF1A,ANXA1
	CHEMOKINE_RECEPTOR_BINDING	26	3.359E-13	CXCL1,CXCL11,CXCL10
	NEGATIVE_REGULATION_OF_CELLULAR_PROCESS	437	5.369E-12	ADAMTS1,SPHK1,HSPB1,IFITM1,TIMP1,ARHGDIB,ANXA1,ANXA5,FST,RND1,S100A11,TAX1BP3,CXCL1,GMNN,CAPG,EMP3,GSN,BTG2
	CELL_DEVELOPMENT	421	7.492E-12	SPHK1,HSPB1,ADORA2A,HSPA2,ANXA1,ANXA5,S100A4,RND1,LGALS1,CD14
	NEGATIVE_REGULATION_OF_BIOLOGICAL_PROCESS	457	1.214E-11	ADAMTS1,SPHK1,HSPB1,IFITM1,TIMP1,ARHGDIB,ANXA1,ANXA5,FST,RND1,S100A11,TAX1BP3,CXCL1,GMNN,CAPG,EMP3,GSN,BTG2
	CELL_PROLIFERATION_GO_0008283	364	1.864E-11	ADAMTS1,SPHK1,CXCL10,IFITM1,TIMP1,FOSL1,TACSTD2,S100A11,CXCL1,EMP3,EMP1,BTG2
	POSITIVE_REGULATION_OF_CELL_PROLIFERATION	110	2.715E-11	CXCL10,TIMP1,SPHK1,FOSL1
	IMMUNE_SYSTEM_PROCESS	228	3.115E-11	IFITM3,ARHGDIB,APLN,PSMB10
	INFLAMMATORY_RESPONSE	96	3.487E-11	S100A8,CXCL11,CXCL10,ADORA2A,ELF3,CXCL1,TNFRSF1A,ANXA1
	CALCIUM_INDEPENDENT_CELL_CELL_ADHESION	16	5.57E-11	CLDN19,CLDN4,CLDN3,CLDN1
	G_PROTEIN_COUPLED_RECEPTOR_BINDING	33	1.167E-10	CXCL11,CXCL10,CXCL1
	REGULATION_OF_DEVELOPMENTAL_PROCESS	312	2.202E-10	SPHK1,HSPB1,RNH1,ANXA1,ANXA5,FST,LGALS1
	CYTOKINE_ACTIVITY	68	2.357E-10	IL19,CXCL11,CXCL10,CXCL1,CNTF
	PROGRAMMED_CELL_DEATH	315	3.975E-10	SPHK1,HSPB1,ADORA2A,ANXA1,ANXA5,LGALS1,CD14
	APOPTOSIS_GO	314	7.604E-10	SPHK1,HSPB1,ADORA2A,ANXA1,ANXA5,LGALS1,CD14
	LOCOMOTORY_BEHAVIOR	70	9.847E-10	CXCL11,CXCL10,FOSL1,CXCL1
	STRUCTURAL_CONSTITUENT_OF_RIBOSOME	72	5.872E-09	
	NUCLEUS	836	7.875E-09	JUN,RANBP1,FOSL1,S100A6,LGALS3,S100A11,NUPR1,EGR1
	NUCLEOBASE__NUCLEOSIDE__NUCLEOTIDE_AND_NUCLEIC_ACID_METABOLIC_PROCESS	779	1.482E-08	GDA,ELF3,ADORA2A,UPP1,FST,KLF5,IRF1,JUNB,FOSL1,S100A11,GADD45G,GMNN,CTPS,EGR1,BTG2,TNFRSF1A
	RESPONSE_TO_STRESS	351	3.307E-08	F5,ELF3,CXCL11,CXCL10,HSPB1,ADORA2A,CLDN3,HSPA2,ANXA1,S100A8,GADD45G,CXCL1,BTG2,TNFRSF1A
	SYSTEM_DEVELOPMENT	595	3.388E-08	GDA,ELF3,CXCL10,ADORA2A,FST,RND1,CXCL1,EMP1,GREM1,SPHK1,COL6A3,RNH1,ANXA2,LAMC2,CSRP3,EGR2
	EXTRACELLULAR_REGION_PART	238	4.97E-08	PVR,MATN1,C1QB,WFDC2,CXCL1,COL6A3,FBLN1,GREM1,FGB,RNH1
	ANATOMICAL_STRUCTURE_DEVELOPMENT	688	5.268E-08	GDA,ELF3,CXCL10,ADORA2A,FST,RND1,CXCL1,EMP1,GREM1,SPHK1,COL6A3,RNH1,ANXA2,LAMC2,CSRP3,EGR2
	POSITIVE_REGULATION_OF_I_KAPPAB_KINASE_NF_KAPPAB_CASCADE	58	7.211E-08	HMOX1,TNFRSF1A,LGALS1
	NON_MEMBRANE_BOUND_ORGANELLE	359	7.882E-08	JUN,JUNB,ARPC1B,HSPB1,ARPC3,ARHGDIB,ANXA1,CAPG,GSN
	INTRACELLULAR_NON_MEMBRANE_BOUND_ORGANELLE	359	7.882E-08	JUN,JUNB,ARPC1B,HSPB1,ARPC3,ARHGDIB,ANXA1,CAPG,GSN
	REGULATION_OF_I_KAPPAB_KINASE_NF_KAPPAB_CASCADE	60	8.174E-08	HMOX1,TNFRSF1A,LGALS1
	NEGATIVE_REGULATION_OF_CELL_PROLIFERATION	117	8.689E-08	ADAMTS1,EMP3,IFITM1,S100A11,BTG2,CXCL1
	MACROMOLECULAR_COMPLEX	598	1.069E-07	ARPC3,C1QB,CAPG,ARPC1B,FGB,RNH1
	MULTICELLULAR_ORGANISMAL_DEVELOPMENT	711	1.246E-07	GDA,ELF3,CXCL10,ADORA2A,ARHGDIB,FST,RND1,CXCL1,EMP1,GREM1,SPHK1,COL6A3,TNFRSF12A,RNH1,ANXA2,LAMC2,CSRP3,EGR2
	IMMUNE_RESPONSE	161	1.559E-07	IFITM3,ARHGDIB,APLN,PSMB10
	REGULATION_OF_PROGRAMMED_CELL_DEATH	246	1.569E-07	LGALS1,SPHK1,HSPB1,ANXA1,ANXA5
	POSITIVE_REGULATION_OF_SIGNAL_TRANSDUCTION	80	2.015E-07	LGALS1,HMOX1,TNFRSF1A
	REGULATION_OF_APOPTOSIS	245	2.925E-07	LGALS1,SPHK1,HSPB1,ANXA1,ANXA5
	EXTRACELLULAR_REGION	320	5.008E-07	WFDC2,COL6A3,FGB,RNH1,PVR,MATN1,C1QB,CXCL1,FBLN1,GREM1,GSN
	RESPONSE_TO_WOUNDING	142	6.918E-07	S100A8,CXCL11,CXCL10,F5,ADORA2A,ELF3,CXCL1,TNFRSF1A,ANXA1
	RNA_METABOLIC_PROCESS	507	7.449E-07	ELF3,FST,KLF5,IRF1,JUNB,FOSL1,TNFRSF1A
	CELL_CELL_ADHESION	54	9.094E-07	CLDN4,CLDN3,CLDN1,CLDN19
	TRANSLATION	119	1.275E-06	HSPB1
	TIGHT_JUNCTION	22	1.415E-06	CLDN19,CLDN4,CLDN3,CLDN1
	APICAL_JUNCTION_COMPLEX	23	2.768E-06	CLDN19,CLDN4,CLDN3,CLDN1
	APICOLATERAL_PLASMA_MEMBRANE	23	2.768E-06	CLDN19,CLDN4,CLDN3,CLDN1
	RESPONSE_TO_EXTERNAL_STIMULUS	228	2.846E-06	S100A8,F5,ELF3,CXCL1,CXCL11,CXCL10,ADORA2A,FOSL1,TNFRSF1A,ANXA1
	NEGATIVE_REGULATION_OF_DEVELOPMENTAL_PROCESS	146	4.685E-06	SPHK1,HSPB1,ANXA1,ANXA5
	CELLULAR_DEFENSE_RESPONSE	35	5.187E-06	FOSL1,ADORA2A
	EXTRACELLULAR_SPACE	173	5.585E-06	PVR,C1QB,WFDC2,CXCL1,FBLN1,GREM1,FGB
	I_KAPPAB_KINASE_NF_KAPPAB_CASCADE	71	5.842E-06	LGALS1,HMOX1,TNFRSF1A
	PROTEASOME_COMPLEX	18	6.612E-06	
	BEHAVIOR	118	7.128E-06	CXCL11,CXCL10,FOSL1,CXCL1
	POSITIVE_REGULATION_OF_DEVELOPMENTAL_PROCESS	150	7.776E-06	FST,SPHK1
	REGULATION_OF_SIGNAL_TRANSDUCTION	137	9.313E-06	LGALS1,TAX1BP3,HMOX1,TNFRSF1A

	NEGATIVE_REGULATION_OF_PROGRAMMED_CELL_DEATH	113	9.353E-06	HSPB1,SPHK1,ANXA1,ANXA5
	ACTIN_FILAMENT_BASED_PROCESS	74	1.005E-05	RND1,ARHGDIB,GSN,CXCL1,CAPG
	PROTEIN_COMPLEX	526	1.09E-05	ARPC3,C1QB,CAPG,ARPC1B,FGB,RNH1
	INTRACELLULAR_SIGNALING_CASCADE	452	1.168E-05	SPHK1,RRAD,ARHGDIB,HMOX1,LGALS1,TAX1BP3,GADD45G,CXCL1,TNFRSF1A
	PHAGOCYTOSIS	12	1.256E-05	ADORA2A,CD14
	RRNA_METABOLIC_PROCESS	10	1.263E-05	
	HUMORAL_IMMUNE_RESPONSE	20	1.276E-05	PSMB10
	NUCLEAR_PART	318	1.306E-05	JUN
	ORGAN_DEVELOPMENT	385	1.349E-05	SPHK1,ELF3,COL6A3,CXCL10,RNH1,ANXA2,FST,LAMC2,CSRP3,EMP1,EGR2
	NEGATIVE_REGULATION_OF_APOPTOSIS	112	2.009E-05	HSPB1,SPHK1,ANXA1,ANXA5
	ACTIN_CYTOSKELETON_ORGANIZATION_AND_BIOGENESIS	67	2.433E-05	RND1,ARHGDIB,GSN,CXCL1,CAPG
	IMMUNE_SYSTEM_DEVELOPMENT	63	2.684E-05	
	ION_BINDING	173	2.975E-05	S100A4,GDA,S100A8,S100A1,CRIP2,SPHK1,GSN,SPOCK2,ANXA1
	RIBOSOME_BIOGENESIS_AND_ASSEMBLY	11	3.299E-05	
	CELLULAR_MACROMOLECULE_METABOLIC_PROCESS	749	3.555E-05	CAPG,WFDC2,TSPAN8,HSPB1,TIMP1,ANXA1,ABCG1,GSN
	CELLULAR_PROTEIN_METABOLIC_PROCESS	740	3.674E-05	CAPG,WFDC2,TSPAN8,HSPB1,TIMP1,ANXA1,ABCG1,GSN
	CELL_SURFACE_RECEPTOR_LINKED_SIGNAL_TRANSDUCTION_GO_0007166	468	3.815E-05	ADAMTS1,SPHK1,RASD1,CXCL10,IFITM1,ANXA1,TACSTD2,TAX1BP3,CXCL1,CD14,TNFRSF1A
	PROTEIN_KINASE_CASCADE	193	3.947E-05	LGALS1,GADD45G,HMOX1,TNFRSF1A
Down (6hr)	ORGANIC_ACID_METABOLIC_PROCESS	130	6.383E-26	SLC7A9,FAH,DDAH1,GATM,QDPR,GCLC,PAH,HGD,TST,SLC25A15,HAO2,NFS1,FTCD,GLYAT,GAMT,CYP4F2
	CARBOXYLIC_ACID_METABOLIC_PROCESS	128	1.221E-23	SLC7A9,FAH,DDAH1,GATM,QDPR,GCLC,PAH,HGD,SLC25A15,HAO2,NFS1,FTCD,GLYAT,GAMT,CYP4F2
	AMINO_ACID_AND_DERIVATIVE_METABOLIC_PROCESS	80	1.185E-22	SLC7A9,FAH,DDAH1,GATM,QDPR,SULT1B1,CCBL1,GCLC,PAH,HGD,DIO1,SLC25A15,NFS1,GAMT
	OXIDOREDUCTASE_ACTIVITY	203	1.495E-18	ALDH8A1,GCDH,HGD,GPX3,SARDH,GRHPR,HAO2,FMO1,HAAO,MIOX,NQO2,QDPR,PAH,KMO,DIO1,CAT,ALDH9A1,DMGDH,NOX4,CYP4F2
	NITROGEN_COMPOUND_METABOLIC_PROCESS	115	4.181E-18	SLC7A9,FAH,DDAH1,GATM,QDPR,SULT1B1,GCLC,PAH,HGD,TST,DIO1,SLC25A15,NFS1,SULT1C2,GAMT
	AMINE_METABOLIC_PROCESS	107	6.477E-18	SLC7A9,FAH,DDAH1,GATM,QDPR,SULT1B1,GCLC,PAH,HGD,DIO1,SLC25A15,NFS1,SULT1C2,GAMT
	ANION_TRANSMEMBRANE_TRANSPORTER_ACTIVITY	42	7.31E-18	SLC22A6,SLC4A1,SLC4A4,SLC22A7,SLC22A8,SLC34A1,SLC34A3,SLC26A4,SLC12A3,SLC17A3,SLC17A1,SLC13A2
	MITOCHONDRION	217	3.191E-17	GATM,GCDH,HMGCS2,NIPSNAP1,SARDH,NFS1,AASS,AMACR,SLC25A15,PC,OAT,DMGDH,GLYAT
	AMINO_ACID_METABOLIC_PROCESS	61	1.543E-16	SLC7A9,FAH,DDAH1,QDPR,GCLC,NFS1,PAH,HGD,SLC25A15
	ANION_TRANSPORT	23	5.621E-15	SLC22A6,SLC4A1,SLC22A7,SLC22A8,SLC34A1,SLC34A3,SLC26A4,SLC17A5,SLC17A1
	NITROGEN_COMPOUND_CATABOLIC_PROCESS	26	2.106E-13	FAH,DDAH1,HGD,TST
	SECONDARY_ACTIVE_TRANSMEMBRANE_TRANSPORTER_ACTIVITY	35	4.124E-13	SLC5A2,SLC15A2,SLC4A4,SLC12A3,SLC22A7,SLC34A3,SLC17A5,SLC17A3,SLC13A2
	SYMPORTER_ACTIVITY	26	7.689E-13	SLC5A2,SLC15A2,SLC4A4,SLC12A3,SLC34A3,SLC17A5,SLC17A3,SLC13A2
	ANION_CATION_SYMPORTER_ACTIVITY	11	7.868E-13	SLC12A3,SLC34A3,SLC17A3,SLC13A2,SLC4A4
	AMINO_ACID_CATABOLIC_PROCESS	22	1.062E-11	FAH,DDAH1,HGD
	SUBSTRATE_SPECIFIC_TRANSMEMBRANE_TRANSPORTER_ACTIVITY	277	3.462E-11	SLC15A2,SLC23A1,SLC22A6,SLC22A2,SLC22A7,SLC22A8,CACNG5,SLC26A4,SLC22A12,SLC7A9,SLC4A1,SLC4A4,SLC34A1,SLC34A3,SLC38A3,SLC13A2,SLC25A15,SLC5A2,SLC37A4,SLC12A3,SLC17A5,SLC17A3,SLC17A1
	INORGANIC_ANION_TRANSMEMBRANE_TRANSPORTER_ACTIVITY	16	9.168E-11	SLC34A1,SLC34A3,SLC26A4,SLC17A3,SLC17A1,SLC4A4
	COFACTOR_METABOLIC_PROCESS	36	2.826E-10	UGT1A1,GCLC,NFS1,FTCD,GLYAT
	AMINE_CATABOLIC_PROCESS	24	3.33E-10	FAH,DDAH1,HGD
	TRANSMEMBRANE_TRANSPORTER_ACTIVITY	301	3.925E-10	ABCG2,SLC15A2,SLC23A1,SLC22A6,SLC22A2,SLC22A7,SLC22A8,CACNG5,SLC26A4,SLC22A12,SLC7A9,SLC4A1,SLC4A4,SLC34A1,SLC34A3,SLC38A3,SLC13A2,SLC25A15,SLC5A2,SLC37A4,SLC12A3,SLC17A5,SLC17A3,SLC17A1
	COENZYME_BINDING	12	2.696E-09	NOX4,GCLC,GRHPR
	SUBSTRATE_SPECIFIC_TRANSPORTER_ACTIVITY	304	3.513E-09	SLC23A1,SLC22A6,SLC22A2,SLC22A7,SLC22A8,CACNG5,SLC7A9,SLC34A1,SLC34A3,SLC13A2,SLC12A3,APOM,SLC15A2,SLC26A4,SLC22A12,SLC4A1,SLC4A4,SLC38A3,SLC25A15,SLC5A2,SLC37A4,SLC17A5,SLC17A3,SLC17A1
	COFACTOR_BINDING	17	6.451E-09	NOX4,GCLC,GRHPR
	PHOSPHATE_TRANSMEMBRANE_TRANSPORTER_ACTIVITY	11	1.338E-08	SLC34A1,SLC34A3,SLC17A3,SLC17A1
	ACTIVE_TRANSMEMBRANE_TRANSPORTER_ACTIVITY	98	2.143E-08	SLC7A9,SLC4A4,SLC34A3,SLC38A3,ABCG2,SLC13A2,SLC25A15,SLC5A2,SLC15A2,SLC22A7,SLC12A3,SLC17A5,SLC17A3
	CELLULAR_CATABOLIC_PROCESS	157	6.035E-08	FAH,DDAH1,HGD,GPX3,STS,HAO2,GBA3,MIOX,TST,UGT1A1,UGT2B4

	ION_TRANSMEMBRANE_TRANSPORTER_ACTIVITY	224	6.041E-08	SLC15A2,SLC22A6,SLC22A2,SLC22A7,SLC22A8,CACNG5,SLC26A4,SLC4A1,SLC4A4,SLC34A1,SLC34A3,SLC13A2,SLC5A2,SLC12A3,SLC17A5,SLC17A3,SLC17A1
	AMINO_ACID_DERIVATIVE_METABOLIC_PROCESS	19	9.316E-08	CCBL1,GATM,SULT1B1,GAMT,DIO1
	LYASE_ACTIVITY	53	9.9E-08	CA3,ACMSD,PCK1,CBS
	GENERATION_OF_PRECURSOR_METABOLITES_AND_ENERGY	92	1.247E-07	APOM,GRHPR,ALDH9A1,NOX4
	CATABOLIC_PROCESS	165	1.481E-07	FAH,DDAH1,HGD,GPX3,STS,HAO2,GBA3,MIOX,TST,UGT1A1,UGT2B4
	OXIDOREDUCTASE_ACTIVITY__ACTING_ON_THE_CH_CH_GROUP_OF_DONORS	19	2.365E-07	GCDH
	CARBOHYDRATE_TRANSMEMBRANE_TRANSPORTER_ACTIVITY	14	2.859E-07	SLC5A2,SLC37A4,SLC17A5
	HYDRO_LYASE_ACTIVITY	17	5.886E-07	CA3,CBS
	COENZYME_METABOLIC_PROCESS	24	7.509E-07	GCLC,FTCD,GLYAT
	SUGAR_TRANSMEMBRANE_TRANSPORTER_ACTIVITY	11	1.124E-06	SLC5A2,SLC17A5,SLC37A4
	HORMONE_METABOLIC_PROCESS	25	1.477E-06	ALDH8A1,UGT1A1,SULT1B1,UGT2B4,DIO1,ALDH9A1
	EXOPEPTIDASE_ACTIVITY	25	1.874E-06	ANPEP,ENPEP,ZMPSTE24,DPP4
	CARBOHYDRATE_METABOLIC_PROCESS	125	2.27E-06	MIOX,FBP1,SLC5A2,GBA3
	GLUTAMINE_FAMILY_AMINO_ACID_METABOLIC_PROCESS	11	2.954E-06	GCLC,DDAH1
	ELECTRON_CARRIER_ACTIVITY	55	5.475E-06	SARDH,NQO2,QDPR,KMO,HAAO,DMGDH,NOX4
	OXIDOREDUCTASE_ACTIVITY__ACTING_ON_THE_ALDEHYDE_OR_OXO_GROUP_OF_DONORS	15	8.095E-06	ALDH8A1,ALDH9A1
	AROMATIC_COMPOUND_METABOLIC_PROCESS	20	1.266E-05	FAH,QDPR,FTCD,HGD,SULT1B1
	FATTY_ACID_METABOLIC_PROCESS	38	2.274E-05	HAO2,GLYAT,CYP4F2
	CELLULAR_LIPID_CATABOLIC_PROCESS	24	2.292E-05	STS,HAO2,UGT2B4
	INORGANIC_ANION_TRANSPORT	14	2.469E-05	SLC34A1,SLC34A3,SLC26A4,SLC17A1
	ELECTRON_TRANSPORT_GO_0006118	34	2.958E-05	GRHPR,ALDH9A1,NOX4
	MONOCARBOXYLIC_ACID_METABOLIC_PROCESS	57	3.819E-05	HAO2,FTCD,GLYAT,CYP4F2
Up (24hr)	NON_MEMBRANE_BOUND_ORGANELLE	359	2.191E-17	TPX2,CCNB2,PRC1,ARPC1B,HMGB2,ARHGDIB,KIF23,KIF22,DLG7,VIM,RFC3,CDC20,CAPG,GSN
	INTRACELLULAR_NON_MEMBRANE_BOUND_ORGANELLE	359	2.191E-17	TPX2,CCNB2,PRC1,ARPC1B,HMGB2,ARHGDIB,KIF23,KIF22,DLG7,VIM,RFC3,CDC20,CAPG,GSN
	NUCLEUS	836	2.969E-15	AIF1,PRC1,KIF22,RANBP1,IRF7,CCNA2,TPX2,HMGB2,DLG7,S100A6,LGALS3,S100A11,NUPR1,SNRPA,NRM
	MITOTIC_CELL_CYCLE	106	6.48E-14	PRC1,CCNA2,KIF23,KIF22,DLG7,TPX2,UBE2C,CDKN3
	CELL_CYCLE_GO_0007049	210	5.26E-13	AIF1,CCNA2,CDC20,TPX2,GMNN,CDKN3,PRC1,KIF23,KIF22,DLG7,UBE2C,HSPA2
	STRUCTURAL_MOLECULE_ACTIVITY	162	5.49E-13	MATN1,FBLN1,ARPC1B,VIM
	CELL_CYCLE_PROCESS	134	3.321E-12	CCNA2,TPX2,CDKN3,PRC1,KIF23,KIF22,DLG7,UBE2C,HSPA2
	REGULATION_OF_CELL_PROLIFERATION	227	9.96E-12	AIF1,ADAMTS1,S100A11,CDKN3,CXCL10,EMP3,TIMP1,BTG2
	CELL_PROLIFERATION_GO_0008283	364	5.234E-11	AIF1,ADAMTS1,TPX2,CXCL10,TIMP1,TACSTD2,S100A11,CDKN3,EMP3,BTG2
	CHROMOSOMAL_PART	60	9.553E-11	KIF22,RFC3
	NUCLEOBASE__NUCLEOSIDE__NUCLEOTIDE_AND_NUCLEIC_ACID_METABOLIC_PROCESS	779	2.853E-10	FST,KLF5,IRF7,HMGB2,PRIM1,RFC3,S100A11,GMNN,CTPS,BTG2
	CHROMOSOME	77	3.511E-10	RFC3,HMGB2,KIF22
	IMMUNE_SYSTEM_PROCESS	228	3.986E-10	RAB3D,GBP2,IFITM3,ARHGDIB,CD83,PSMB10
	POSITIVE_REGULATION_OF_BIOLOGICAL_PROCESS	461	6.248E-10	CXCL10,DAP,TIMP1,FST,LGALS1,MX1,UBE2C
	POSITIVE_REGULATION_OF_CELLULAR_PROCESS	436	8.927E-10	CXCL10,DAP,TIMP1,LGALS1,MX1,UBE2C
	REPLICATION_FORK	13	9.262E-10	RFC3
	DEFENSE_RESPONSE	175	1.485E-09	AIF1,LGALS3BP,CXCL9,CXCL11,CXCL10,MX1,CD83
	DNA_METABOLIC_PROCESS	170	3.41E-09	PRIM1,RFC3,S100A11,GMNN,HMGB2,BTG2
	SPINDLE	30	3.69E-09	KIF23,PRC1,DLG7,TPX2,CDC20
	NUCLEAR_PART	318	4.133E-09	HMGB2,SNRPA,NRM
	CYTOSKELETON	210	5.778E-09	CDC20,TPX2,CCNB2,CAPG,PRC1,ARPC1B,ARHGDIB,KIF23,DLG7,GSN,VIM
	CHEMOKINE_ACTIVITY	25	7.351E-09	CXCL9,CXCL11,CXCL10
	MACROMOLECULAR_COMPLEX	598	9.394E-09	KIF23,KIF22,C1QB,CAPG,ARPC1B,FGB,RFC3,SNRPA
	CYTOSKELETAL_PART	132	1.029E-08	CDC20,TPX2,CAPG,PRC1,ARPC1B,KIF23,DLG7,VIM
	M_PHASE_OF_MITOTIC_CELL_CYCLE	59	1.369E-08	KIF22,DLG7,CCNA2,TPX2,UBE2C
	CELL_CYCLE_PHASE	119	1.374E-08	CCNA2,KIF22,DLG7,TPX2,UBE2C,HSPA2,CDKN3
	CHEMOKINE_RECEPTOR_BINDING	26	1.622E-08	CXCL9,CXCL11,CXCL10
	CELL_DEVELOPMENT	421	1.949E-08	DAP,HSPA2,ANXA5,ANXA4,S100A4,LGALS1,RAB3D,CD14,MX1
	POSITIVE_REGULATION_OF_CELL_PROLIFERATION	110	2.029E-08	CXCL10,TIMP1
	MICROTUBULE_CYTOSKELETON	93	3.215E-08	PRC1,CDC20,KIF23,DLG7,TPX2,CCNB2
	NEGATIVE_REGULATION_OF_CELLULAR_PROCESS	437	3.792E-08	AIF1,ADAMTS1,TIMP1,ARHGDIB,ANXA5,ANXA4,FST,S100A11,GMNN,CDKN3,CAPG,EMP3,GSN,BTG2,IRF7
	M_PHASE	80	4.016E-08	CCNA2,KIF22,DLG7,TPX2,UBE2C,HSPA2
	REGULATION_OF_DEVELOPMENTAL_PROCESS	312	7.157E-08	DAP,ANXA5,ANXA4,FST,LGALS1,MX1
	MITOSIS	57	8.797E-08	KIF22,CCNA2,TPX2,UBE2C
	NEGATIVE_REGULATION_OF_BIOLOGICAL_PROCESS	457	8.842E-08	AIF1,ADAMTS1,TIMP1,ARHGDIB,ANXA5,ANXA4,FST,S100A11,GMNN,CDKN3,CAPG,EMP3,GSN,BTG2,IRF7
	STRUCTURAL_CONSTITUENT_OF_RIBOSOME	72	1.117E-07	
	RNA_METABOLIC_PROCESS	507	1.346E-07	FST,KLF5,IRF7,HMGB2,PRIM1
	CALCIUM_INDEPENDENT_CELL_CELL_ADHESION	16	1.785E-07	CLDN4

	RESPONSE_TO_DNA_DAMAGE_STIMULUS	103	2.286E-07	CCNA2,RFC3,HMGB2,BTG2
	DNA_REPLICATION	67	2.878E-07	PRIM1,RFC3,HMGB2,S100A11,GMNN
	PROGRAMMED_CELL_DEATH	315	2.916E-07	DAP,ANXA5,ANXA4,LGALS1,CD14,MX1
	CYTOSKELETON_ORGANIZATION_AND_BIOGENESIS	129	3.438E-07	CAPG,PRC1,ARHGDIB,KIF23,GSN
	APOPTOSIS_GO	314	4.824E-07	DAP,ANXA5,ANXA4,LGALS1,CD14,MX1
	RESPONSE_TO_STRESS	351	5.868E-07	AIF1,CCNA2,CXCL11,CXCL10,HMGB2,HSPA2,RFC3,CXCL9,BTG2
	PROTEIN_COMPLEX	526	6.467E-07	KIF23,KIF22,C1QB,CAPG,ARPC1B,FGB,RFC3
	CELLULAR_MACROMOLECULE_METABOLIC_PROCESS	749	1.054E-06	CAPG,UBE2C,WFDC2,TSPAN8,TIMP1,CDC20,ABCG1,GSN,PTPRB
	CELLULAR_PROTEIN_METABOLIC_PROCESS	740	1.405E-06	CAPG,UBE2C,WFDC2,TSPAN8,TIMP1,CDC20,ABCG1,GSN,PTPRB
	INFLAMMATORY_RESPONSE	96	1.693E-06	AIF1,CXCL11,CXCL10,CXCL9
	INTRACELLULAR_ORGANELLE_PART	705	2.07E-06	PRC1,KIF23,KIF22,CAPG,TPX2,ARPC1B,HMGB2,DLG7,VIM,RFC3,CDC20,SNRPA,NRM
	PROTEIN_METABOLIC_PROCESS	809	2.255E-06	MATN1,GJA4,CAPG,UBE2C,WFDC2,TSPAN8,TIMP1,CDC20,ABCG1,GSN,PTPRB
	DNA_REPAIR	82	2.382E-06	RFC3,HMGB2,BTG2
	G_PROTEIN_COUPLED_RECEPTOR_BINDING	33	2.604E-06	CXCL11,CXCL10,CXCL9
	ORGANELLE_PART	709	3.296E-06	PRC1,KIF23,KIF22,CAPG,TPX2,ARPC1B,HMGB2,DLG7,VIM,RFC3,CDC20,SNRPA,NRM
	LOCOMOTORY_BEHAVIOR	70	3.616E-06	CXCL11,CXCL10,CXCL9
	IMMUNE_RESPONSE	161	5.125E-06	GBP2,IFITM3,ARHGDIB,CD83,PSMB10
	ORGANELLE_ORGANIZATION_AND_BIOGENESIS	290	5.69E-06	PRC1,ARHGDIB,KIF23,CAPG,GSN
	REGULATION_OF_PROGRAMMED_CELL_DEATH	246	5.757E-06	LGALS1,DAP,MX1,ANXA5,ANXA4
	ACTIN_FILAMENT_BASED_PROCESS	74	6.611E-06	ARHGDIB,GSN,CAPG
	DNA_DEPENDENT_DNA_REPLICATION	37	6.695E-06	PRIM1,RFC3,HMGB2,GMNN,S100A11
	CELLULAR_DEFENSE_RESPONSE	35	7.619E-06	CXCL9,LGALS3BP
	HUMORAL_IMMUNE_RESPONSE	20	8.847E-06	PSMB10,CD83
	REGULATION_OF_APOPTOSIS	245	9.561E-06	LGALS1,DAP,MX1,ANXA5,ANXA4
	RESPONSE_TO_ENDOGENOUS_STIMULUS	129	1.032E-05	CCNA2,RFC3,HMGB2,BTG2
	TRANSLATION	119	1.433E-05	
	POSITIVE_REGULATION_OF_I_KAPPAB_KINASE_NF_KAPPAB_CASCADE	58	1.795E-05	LGALS1
	IMMUNE_SYSTEM_DEVELOPMENT	63	1.917E-05	RAB3D
	ACTIN_CYTOSKELETON_ORGANIZATION_AND_BIOGENESIS	67	2.096E-05	ARHGDIB,GSN,CAPG
	REGULATION_OF_I_KAPPAB_KINASE_NF_KAPPAB_CASCADE	60	2.411E-05	LGALS1
	RNA_BIOSYNTHETIC_PROCESS	396	3.299E-05	HMGB2,FST,PRIM1,KLF5,IRF7
	SYSTEM_DEVELOPMENT	595	3.455E-05	CXCL10,FST,RAB3D,GREM1,ANXA2,LAMC2,CSRP3
	NEGATIVE_REGULATION_OF_CELL_PROLIFERATION	117	3.495E-05	AIF1,ADAMTS1,EMP3,S100A11,BTG2,CDKN3
	CYTOKINE_ACTIVITY	68	3.616E-05	IL19,CXCL11,CXCL10,CXCL9,CNTF
	NUCLEAR_CHROMOSOME	32	3.935E-05	HMGB2
Down (24hr)	ORGANIC_ACID_METABOLIC_PROCESS	130	5.677E-30	SLC7A9,FAH,DDAH1,GATM,QDPR,MPST,GCLC,PAH,HGD,TST,SLC25A15,HPD,HAO2,NFS1,FTCD,GLYAT,GAMT,CYP4F2
	CARBOXYLIC_ACID_METABOLIC_PROCESS	128	5.278E-27	SLC7A9,FAH,DDAH1,GATM,QDPR,GCLC,PAH,HGD,SLC25A15,HPD,HAO2,NFS1,FTCD,GLYAT,GAMT,CYP4F2
	AMINO_ACID_AND_DERIVATIVE_METABOLIC_PROCESS	80	6.501E-27	SLC7A9,FAH,DDAH1,GATM,QDPR,SULT1B1,CCBL1,GCLC,PAH,HGD,DIO1,SLC25A15,HPD,NFS1,GAMT
	OXIDOREDUCTASE_ACTIVITY	203	9.092E-25	ALDH8A1,GPD1,GCDH,HGD,GPX3,GRHPR,HAO2,FMO1,HAAO,MIOX,AKR7A2,AKR7A3,NQO2,QDPR,PAH,KMO,DIO1,CAT,ALDH9A1,DMGDH,NOX4,CYP4F2
	NITROGEN_COMPOUND_METABOLIC_PROCESS	115	9.672E-25	SLC7A9,FAH,DDAH1,GATM,QDPR,SULT1B1,MPST,GCLC,PAH,HGD,TST,DIO1,SLC25A15,HPD,NFS1,SULT1C2,GAMT
	AMINE_METABOLIC_PROCESS	107	3.122E-23	SLC7A9,FAH,DDAH1,GATM,QDPR,SULT1B1,GCLC,PAH,HGD,DIO1,SLC25A15,HPD,NFS1,SULT1C2,GAMT
	MITOCHONDRION	217	3.394E-21	GATM,TBRG4,ABAT,GCDH,HMGCS2,NIPSNAP1,NFS1,AMACR,SLC25A15,PC,OAT,DMGDH,GLYAT
	ANION_TRANSMEMBRANE_TRANSPORTER_ACTIVITY	42	1.263E-20	SLC22A6,SLC4A4,SLC22A7,SLC22A8,SLC34A1,SLC34A3,SLC26A4,SLC12A3,SLC17A3,SLC17A1,SLC13A2
	AMINO_ACID_METABOLIC_PROCESS	61	1.668E-19	SLC7A9,FAH,DDAH1,HPD,QDPR,GCLC,NFS1,PAH,HGD,SLC25A15
	SUBSTRATE_SPECIFIC_TRANSMEMBRANE_TRANSPORTER_ACTIVITY	277	3.821E-16	AQP7,SLC15A2,SLC23A1,SLC22A6,SLC22A2,SLC22A7,SLC22A8,CACNG5,SLC26A4,SLC22A12,SLC7A9,SLC2A5,SLC4A4,SLC34A1,SLC34A3,SLC38A3,SLC13A2,SLC25A15,SLC5A2,SLC12A3,SLC17A5,SLC17A3,SLC17A1
	ANION_TRANSPORT	23	5.296E-16	SLC22A6,SLC22A7,SLC22A8,SLC34A1,SLC34A3,SLC26A4,SLC17A5,SLC17A1
	NITROGEN_COMPOUND_CATABOLIC_PROCESS	26	5.729E-15	FAH,DDAH1,HPD,HGD,TST,MPST
	SYMPORTER_ACTIVITY	26	1.831E-14	SLC5A2,SLC15A2,SLC4A4,SLC12A3,SLC34A3,SLC17A5,SLC17A3,SLC13A2
	TRANSMEMBRANE_TRANSPORTER_ACTIVITY	301	2.749E-14	AQP7,ABCG2,SLC15A2,SLC23A1,SLC22A6,SLC22A2,SLC22A7,SLC22A8,CACNG5,SLC26A4,SLC22A12,SLC7A9,SLC2A5,SLC4A4,SLC34A1,SLC34A3,SLC38A3,SLC13A2,SLC25A15,SLC5A2,SLC12A3,SLC17A5,SLC17A3,SLC17A1
	ANION_CATION_SYMPORTER_ACTIVITY	11	4.476E-14	SLC12A3,SLC34A3,SLC17A3,SLC13A2,SLC4A4
	SECONDARY_ACTIVE_TRANSMEMBRANE_TRANSPORTER_ACTIVITY	35	6.976E-14	SLC5A2,SLC15A2,SLC4A4,SLC12A3,SLC22A7,SLC34A3,SLC17A5,SLC17A3,SLC13A2

	SUBSTRATE_SPECIFIC_TRANSPORTER_ACTIVITY	304	2.38E-13	SLC23A1,SLC22A6,SLC22A2,SLC22A7,SLC22A8,CACNG5,SLC7A9,SLC2A5,SLC34A1,SLC34A3,SLC13A2,SLC12A3,AQP7,APOM,SLC15A2,SLC26A4,SLC22A12,SLC4A4,SLC38A3,SLC25A15,SLC5A2,SLC17A5,SLC17A3,SLC17A1
	INORGANIC_ANION_TRANSMEMBRANE_TRANSPORTER_ACTIVITY	16	1.052E-12	SLC34A1,SLC34A3,SLC26A4,SLC17A3,SLC17A1,SLC4A4
	AMINO_ACID_CATABOLIC_PROCESS	22	1.991E-12	FAH,DDAH1,HPD,HGD
	AMINE_CATABOLIC_PROCESS	24	7.546E-11	FAH,DDAH1,HPD,HGD
	COFACTOR_METABOLIC_PROCESS	36	1.212E-10	UGT1A1,GCLC,NFS1,FTCD,GLYAT
	PHOSPHATE_TRANSMEMBRANE_TRANSPORTER_ACTIVITY	11	2.065E-10	SLC34A1,SLC34A3,SLC17A3,SLC17A1
	HORMONE_METABOLIC_PROCESS	25	2.47E-10	ALDH8A1,UGT1A1,SULT1B1,UGT2B4,DIO1,ALDH9A1
	ACTIVE_TRANSMEMBRANE_TRANSPORTER_ACTIVITY	98	3.433E-10	SLC7A9,SLC4A4,SLC34A3,SLC38A3,ABCG2,SLC13A2,SLC25A15,SLC5A2,SLC15A2,SLC22A7,SLC12A3,SLC17A5,SLC17A3
	ION_TRANSMEMBRANE_TRANSPORTER_ACTIVITY	224	3.551E-10	SLC15A2,SLC22A6,SLC22A2,SLC22A7,SLC22A8,CACNG5,SLC26A4,SLC4A4,SLC34A1,SLC34A3,SLC13A2,SLC5A2,SLC12A3,SLC17A5,SLC17A3,SLC17A1
	AMINO_ACID_DERIVATIVE_METABOLIC_PROCESS	19	7.957E-10	CCBL1,GATM,SULT1B1,GAMT,DIO1
	COFACTOR_BINDING	17	6.842E-09	NOX4,GCLC,GRHPR,ABAT
	COENZYME_BINDING	12	8.203E-09	NOX4,GCLC,GRHPR
	CARBOHYDRATE_TRANSMEMBRANE_TRANSPORTER_ACTIVITY	14	8.309E-09	SLC5A2,SLC17A5,SLC2A5
	CELLULAR_CATABOLIC_PROCESS	157	8.627E-09	FAH,DDAH1,MPST,HGD,GPX3,STS,HAO2,GBA3,MIOX,TST,HPD,UGT1A1,UGT2B4
	ORGANIC_ACID_TRANSPORT	31	1.442E-08	SLC38A3,SLC22A12,SLC7A9,SLC22A6,SLC25A15
	CATABOLIC_PROCESS	165	2.119E-08	FAH,DDAH1,MPST,HGD,GPX3,STS,HAO2,GBA3,MIOX,TST,HPD,UGT1A1,UGT2B4
	GENERATION_OF_PRECURSOR_METABOLITES_AND_ENERGY	92	2.676E-08	AQP7,APOM,QPRT,GRHPR,ALDH9A1,NOX4
	SUGAR_TRANSMEMBRANE_TRANSPORTER_ACTIVITY	11	4.744E-08	SLC5A2,SLC17A5,SLC2A5
	ELECTRON_CARRIER_ACTIVITY	55	5.266E-08	AKR7A2,AKR7A3,NQO2,QDPR,KMO,HAAO,DMGDH,NOX4
	ORGANIC_ACID_TRANSMEMBRANE_TRANSPORTER_ACTIVITY	35	6.068E-08	SLC38A3,SLC22A12,SLC7A9,SLC13A2,SLC25A15
	LYASE_ACTIVITY	53	1.055E-07	CA3,ODC1,ACMSD,PCK1,CBS
	MITOCHONDRIAL_PART	100	5.86E-07	GATM,SLC25A15,NFS1
	L_AMINO_ACID_TRANSMEMBRANE_TRANSPORTER_ACTIVITY	13	5.915E-07	SLC38A3,SLC7A9,SLC25A15
	AROMATIC_COMPOUND_METABOLIC_PROCESS	20	6.436E-07	FAH,HPD,QDPR,FTCD,HGD,SULT1B1
	EXOPEPTIDASE_ACTIVITY	25	6.855E-07	ANPEP,ENPEP,DPP4
	CARBOHYDRATE_METABOLIC_PROCESS	125	7.106E-07	SLC2A5,MIOX,AKR7A2,FBP1,SLC5A2,GBA3
	COENZYME_METABOLIC_PROCESS	24	8.396E-07	GCLC,FTCD,GLYAT
	OXIDOREDUCTASE_ACTIVITY__ACTING_ON_CH_OH_GROUP_OF_DONORS	44	8.818E-07	MIOX,AKR7A2,AKR7A3,GRHPR,HAO2,GPD1
	CELLULAR_LIPID_CATABOLIC_PROCESS	24	1.818E-06	STS,HAO2,UGT2B4
	CARBOXYLIC_ACID_TRANSPORT	30	2.506E-06	SLC38A3,SLC7A9,SLC22A6,SLC25A15
	HYDRO_LYASE_ACTIVITY	17	2.772E-06	CA3,CBS
	GLUTAMINE_FAMILY_AMINO_ACID_METABOLIC_PROCESS	11	2.796E-06	GCLC,DDAH1
	MONOSACCHARIDE_TRANSMEMBRANE_TRANSPORTER_ACTIVITY	10	2.837E-06	SLC5A2,SLC2A5
	VITAMIN_BINDING	10	3.414E-06	GC,ABAT,PC
	FATTY_ACID_METABOLIC_PROCESS	38	4.185E-06	HAO2,GLYAT,CYP4F2
	AMINO_ACID_DERIVATIVE_BIOSYNTHETIC_PROCESS	10	4.26E-06	GATM,GAMT
	LIPID_CATABOLIC_PROCESS	25	4.29E-06	STS,HAO2,UGT2B4
	ELECTRON_TRANSPORT_GO_0006118	34	4.323E-06	GRHPR,ALDH9A1,NOX4
	INORGANIC_ANION_TRANSPORT	14	6.322E-06	SLC34A1,SLC34A3,SLC26A4,SLC17A1
	MONOCARBOXYLIC_ACID_METABOLIC_PROCESS	57	6.537E-06	HAO2,FTCD,GLYAT,CYP4F2
	CARBOXYLIC_ACID_TRANSMEMBRANE_TRANSPORTER_ACTIVITY	34	6.625E-06	SLC38A3,SLC7A9,SLC13A2,SLC25A15
	OXIDOREDUCTASE_ACTIVITY__ACTING_ON_THE_CH_CH_GROUP_OF_DONORS	19	9.033E-06	GCDH
	CATION_TRANSMEMBRANE_TRANSPORTER_ACTIVITY	175	9.869E-06	SLC15A2,SLC22A2,CACNG5,SLC4A4,SLC34A3,SLC13A2,SLC5A2,SLC12A3,SLC17A5,SLC17A3
	OXIDOREDUCTASE_ACTIVITY_GO_0016616	40	2.343E-05	MIOX,AKR7A2,AKR7A3,GRHPR,GPD1
	ION_TRANSPORT	148	2.813E-05	SLC34A1,SLC34A3,SLC22A6,SLC22A2,SLC22A7,SLC22A8,SLC26A4,SLC17A5,SLC17A3,SLC17A1
	OXIDOREDUCTASE_ACTIVITY__ACTING_ON_THE_ALDEHYDE_OR_OXO_GROUP_OF_DONORS	15	3.028E-05	ALDH8A1,ALDH9A1
	MICROBODY	34	3.68E-05	AMACR,PEX11A,HAO2
	PEROXISOME	34	3.68E-05	AMACR,PEX11A,HAO2
	CARBOXY_LYASE_ACTIVITY	14	3.681E-05	ODC1,ACMSD,PCK1
	MITOCHONDRIAL_ENVELOPE	69	3.921E-05	GATM,SLC25A15
	OXYGEN_BINDING	14	3.935E-05	NOX4,CYP1A1,CYP2E1
Up (120hr)	IMMUNE_SYSTEM_PROCESS	228	2.137E-23	CD74,C2,CCL2,IFITM3,CD83
	EXTRACELLULAR_REGION_PART	238	3.109E-22	PVR,C1QB,LGALS3BP,COL18A1,CXCL1,COL3A1,C2,FBLN1,IL1B,MGP,COL1A2,CCL2,GREM1,FGB,EFEMP2
	STRUCTURAL_MOLECULE_ACTIVITY	162	1.376E-20	FBLN1,MGP,EFEMP2
	POSITIVE_REGULATION_OF_BIOLOGICAL_PROCESS	461	2.655E-20	C2,CXCL10,TIMP1,FST,MX1
	POSITIVE_REGULATION_OF_CELLULAR_PROCESS	436	1.388E-19	CXCL10,TIMP1,MX1
	DEFENSE_RESPONSE	175	3.082E-19	LGALS3BP,ELF3,CXCL1,C2,CXCL11,CXCL10,MX1,CD83
	NEGATIVE_REGULATION_OF_CELLULAR_PROCESS	437	3.154E-19	ADAMTS1,HPGD,IL1B,CCL2,TIMP1,FST,CD74,COL18A1,S100A11,CXCL1,BIRC3,CAPG,BTG2,IRF7

NEGATIVE_REGULATION_OF_BIOLOGICAL_PROCESS	457	3.8E-19	ADAMTS1,HPGD,IL1B,CCL2,TIMP1,FST,CD74,COL18A1,S100A11,CXCL1,BIRC3,CAPG,BTG2,IRF7
REGULATION_OF_DEVELOPMENTAL_PROCESS	312	2.016E-18	CCL2,FST,CD74,BIRC3,MX1
CHEMOKINE_ACTIVITY	25	4.336E-18	CXCL1,CCL2,CXCL11,CXCL10
CELL_DEVELOPMENT	421	5.627E-18	IL1B,CCL2,HSPA2,S100A4,CD74,BIRC3,CD14,MX1
PROGRAMMED_CELL_DEATH	315	1.433E-17	IL1B,CCL2,CD74,BIRC3,CD14,MX1
CHEMOKINE_RECEPTOR_BINDING	26	1.848E-17	CXCL1,CCL2,CXCL11,CXCL10
EXTRACELLULAR_REGION	320	2.318E-17	C2,IL1B,MGP,COL1A2,CCL2,FGB,EFEMP2,PVR,C1QB,LGALS3BP,COL18A1,CXCL1,COL3A1,FBLN1,GREM1
APOPTOSIS_GO	314	3.998E-17	IL1B,CCL2,CD74,BIRC3,CD14,MX1
REGULATION_OF_CELL_PROLIFERATION	227	1.4E-16	ADAMTS1,COL18A1,S100A11,CXCL1,CXCL10,IL1B,TIMP1,BTG2
IMMUNE_RESPONSE	161	1.611E-16	CD74,C2,CCL2,IFITM3,CD83
PROTEINACEOUS_EXTRACELLULAR_MATRIX	70	8.469E-16	MGP,COL1A2,COL18A1,EFEMP2,COL3A1,FBLN1
ANATOMICAL_STRUCTURE_DEVELOPMENT	688	5.14E-15	ELF3,CXCL10,MGP,CCL2,FST,COL18A1,CXCL1,GREM1,COL1A2,ANXA2,LAMC2
EXTRACELLULAR_MATRIX	72	6.589E-15	MGP,COL1A2,COL18A1,EFEMP2,COL3A1,FBLN1
CELL_PROLIFERATION_GO_0008283	364	1.318E-14	ADAMTS1,CXCL10,IL1B,TIMP1,TACSTD2,CD74,COL18A1,S100A11,CXCL1,BTG2
INFLAMMATORY_RESPONSE	96	1.673E-14	CXCL11,CXCL10,ELF3,CXCL1,C2
MULTICELLULAR_ORGANISMAL_DEVELOPMENT	711	2.393E-14	ELF3,CXCL10,MGP,CCL2,FST,COL18A1,CXCL1,GREM1,COL1A2,TNFRSF12A,ANXA2,LAMC2
SYSTEM_DEVELOPMENT	595	2.613E-14	ELF3,CXCL10,MGP,CCL2,FST,COL18A1,CXCL1,GREM1,COL1A2,ANXA2,LAMC2
G_PROTEIN_COUPLED_RECEPTOR_BINDING	33	3.356E-14	CXCL11,CXCL10,CXCL1,CCL2
REGULATION_OF_PROGRAMMED_CELL_DEATH	246	8.764E-14	CD74,BIRC3,MX1,CCL2
REGULATION_OF_APOPTOSIS	245	2.427E-13	CD74,BIRC3,MX1,CCL2
CELLULAR_MACROMOLECULE_METABOLIC_PROCESS	749	4.206E-13	C2,CCL2,CAPG,TSPAN8,TIMP1,ABCG1,LOX
PROTEIN_METABOLIC_PROCESS	809	5.435E-13	C2,CCL2,CD74,CAPG,TSPAN8,TIMP1,ABCG1,LOX
CELLULAR_PROTEIN_METABOLIC_PROCESS	740	6.984E-13	C2,CCL2,CAPG,TSPAN8,TIMP1,ABCG1,LOX
COLLAGEN	18	1.618E-12	COL18A1,COL3A1,COL1A2
EXTRACELLULAR_SPACE	173	3.066E-12	PVR,C1QB,LGALS3BP,CXCL1,C2,FBLN1,IL1B,CCL2,GREM1,FGB
LOCOMOTORY_BEHAVIOR	70	1.745E-11	CXCL11,CXCL10,CCL2,CXCL1
INTRACELLULAR_SIGNALING_CASCADE	452	1.807E-11	CCL2,CXCL1
HUMORAL_IMMUNE_RESPONSE	20	2.296E-11	CCL2,C2,CD83
NON_MEMBRANE_BOUND_ORGANELLE	359	2.401E-11	CCNB2,PRC1,CAPG
INTRACELLULAR_NON_MEMBRANE_BOUND_ORGANELLE	359	2.401E-11	CCNB2,PRC1,CAPG
CYTOKINE_ACTIVITY	68	3.3E-11	IL19,CXCL11,CXCL10,CCL2,CXCL1,CNTF
PROTEIN_KINASE_CASCADE	193	3.471E-11	CCL2
REGULATION_OF_I_KAPPAB_KINASE_NF_KAPPAB_CASCADE	60	7.045E-11	
RESPONSE_TO_WOUNDING	142	1.037E-10	CXCL11,CXCL10,ELF3,CXCL1,C2
ORGAN_DEVELOPMENT	385	1.072E-10	ELF3,CXCL10,MGP,COL1A2,CCL2,ANXA2,FST,LAMC2,COL18A1
CYTOSKELETON	210	1.228E-10	CCNB2,CAPG,PRC1
ACTIN_FILAMENT_BASED_PROCESS	74	1.547E-10	CXCL1,CAPG
EXTRACELLULAR_MATRIX_PART	35	1.908E-10	COL1A2,COL3A1,COL18A1,EFEMP2
POSITIVE_REGULATION_OF_I_KAPPAB_KINASE_NF_KAPPAB_CASCADE	58	2.221E-10	
NUCLEUS	836	2.641E-10	PRC1,BIRC3,IRF7,S100A6,LGALS3,S100A11
I_KAPPAB_KINASE_NF_KAPPAB_CASCADE	71	3.086E-10	
CELL_SURFACE_RECEPTOR_LINKED_SIGNAL_TRANSDUCTION_GO_0007166	468	4.498E-10	ADAMTS1,RASD1,HPGD,CXCL10,CCL2,TACSTD2,AKAP12,CXCL1,BIRC3,CD14
ACTIN_CYTOSKELETON_ORGANIZATION_AND_BIOGENESIS	67	4.575E-10	CXCL1,CAPG
NEGATIVE_REGULATION_OF_CELL_PROLIFERATION	117	5.772E-10	IL1B,ADAMTS1,COL18A1,S100A11,BTG2,CXCL1
RESPONSE_TO_EXTERNAL_STIMULUS	228	6.007E-10	ELF3,CXCL1,C2,CXCL11,CXCL10,CCL2
STRUCTURAL_CONSTITUENT_OF_RIBOSOME	72	8.955E-10	
RESPONSE_TO_STRESS	351	9.99E-10	ELF3,C2,CXCL11,CXCL10,HSPA2,CXCL1,BTG2
POSITIVE_REGULATION_OF_DEVELOPMENTAL_PROCESS	150	1.498E-09	FST,MX1
RNA_BIOSYNTHETIC_PROCESS	396	1.628E-09	ELF3,CREM,FST,KLF5,IRF7
TRANSCRIPTION__DNA_DEPENDENT	394	1.781E-09	ELF3,CREM,FST,KLF5,IRF7
POSITIVE_REGULATION_OF_SIGNAL_TRANSDUCTION	80	1.787E-09	
POSITIVE_REGULATION_OF_CELL_PROLIFERATION	110	2.008E-09	CXCL10,TIMP1
IMMUNE_SYSTEM_DEVELOPMENT	63	2.185E-09	
RNA_METABOLIC_PROCESS	507	3.64E-09	ELF3,FST,KLF5,IRF7,CREM
CYTOSKELETON_ORGANIZATION_AND_BIOGENESIS	129	8.016E-09	CXCL1,CAPG,PRC1
REGULATION_OF_SIGNAL_TRANSDUCTION	137	8.18E-09	
HEMOPOIETIC_OR_LYMPHOID_ORGAN_DEVELOPMENT	60	9.59E-09	
TRANSCRIPTION	474	1.919E-08	ELF3,CREM,FST,KLF5,IRF7
TRANSCRIPTION_FROM_RNA_POLYMERASE_II_PROMOTER	292	1.948E-08	ELF3,FST,KLF5,IRF7
ION_BINDING	173	1.964E-08	S100A4,LOX,SPOCK2
EPIDERMIS_DEVELOPMENT	40	2.196E-08	FST,LAMC2
EXTRACELLULAR_MATRIX_STRUCTURAL_CONSTITUENT	20	3.329E-08	EFEMP2,MGP,FBLN1
ECTODERM_DEVELOPMENT	42	4.893E-08	FST,LAMC2
CALCIUM_INDEPENDENT_CELL_CELL_ADHESION	16	5.453E-08	CLDN4
ENZYME_REGULATOR_ACTIVITY	221	5.597E-08	CXCL1,CXCL10,TIMP1,ANXA2
RECEPTOR_BINDING	246	5.774E-08	CXCL1,CNTF,IL19,CXCL11,CXCL10,CCL2
HEMOPOIESIS	58	5.897E-08	
CELL_CELL_ADHESION	54	1.128E-07	CLDN4,MGP
PHAGOCYTOSIS	12	1.549E-07	CD14
CELLULAR_DEFENSE_RESPONSE	35	2.103E-07	LGALS3BP
MITOTIC_CELL_CYCLE	106	4.006E-07	PRC1
NUCLEOBASE__NUCLEOSIDE__NUCLEOTIDE_AND_NUCLEIC_ACID_METABOLIC_PROCESS	779	4.901E-07	ELF3,FST,KLF5,IRF7,CREM,S100A11,BTG2
CELL_CYCLE_GO_0007049	210	5.889E-07	HPGD,PRC1,HSPA2

	CYTOSKELETAL_PART	132	6.062E-07	CAPG,PRC1
	APICAL_JUNCTION_COMPLEX	23	6.335E-07	CLDN4
	APICOLATERAL_PLASMA_MEMBRANE	23	6.335E-07	CLDN4
	ANATOMICAL_STRUCTURE_MORPHOGENESIS	250	6.494E-07	COL18A1,ELF3,CCL2
	POSITIVE_REGULATION_OF_IMMUNE_SYSTEM_PROCESS	33	7.23E-07	C2
	NEGATIVE_REGULATION_OF_DEVELOPMENTAL_PROCESS	146	7.316E-07	CD74,BIRC3,CCL2
	CATION_BINDING	141	8.623E-07	S100A4,LOX,SPOCK2
	ANTI_APOPTOSIS	86	9.648E-07	CCL2,BIRC3
	TISSUE_DEVELOPMENT	84	1.321E-06	FST,MGP,LAMC2
	REGULATION_OF_ACTIN_FILAMENT_LENGTH	10	1.56E-06	CAPG
	NEGATIVE_REGULATION_OF_PROGRAMMED_CELL_DEATH	113	1.782E-06	CCL2,CD74,BIRC3
	TIGHT_JUNCTION	22	1.918E-06	CLDN4
	POSITIVE_REGULATION_OF_MULTICELLULAR_ORGANISMAL_PROCESS	44	1.93E-06	FST,C2
	ACTIN_CYTOSKELETON	73	2.201E-06	CAPG
	ADAPTIVE_IMMUNE_RESPONSE	17	3.41E-06	CD74,C2
	PROTEOLYSIS	129	3.843E-06	TIMP1,C2
	REGULATION_OF_CELLULAR_METABOLIC_PROCESS	475	4.332E-06	FST,CAPG,IRF7,CREM,TIMP1,S100A11
	NEGATIVE_REGULATION_OF_APOPTOSIS	112	4.757E-06	CCL2,CD74,BIRC3
	CALCIUM_ION_BINDING	66	5.435E-06	S100A4,SPOCK2
	REGULATION_OF_METABOLIC_PROCESS	484	6.005E-06	FST,CAPG,IRF7,CREM,TIMP1,S100A11
	REGULATION_OF_IMMUNE_SYSTEM_PROCESS	41	6.905E-06	C2
	LEUKOCYTE_ACTIVATION	40	9.022E-06	
	RESPONSE_TO_BIOTIC_STIMULUS	69	1.109E-05	HSPA2,IRF7
	MACROMOLECULAR_COMPLEX	598	1.169E-05	CACNB3,C1QB,CAPG,FGB
	REGULATION_OF_TRANSCRIPTION__DNA_DEPENDENT	276	1.265E-05	CREM,FST,IRF7
	CELL_DIVISION	14	1.3E-05	PRC1
	BEHAVIOR	118	1.773E-05	CXCL11,CXCL10,CCL2,CXCL1
	MACROMOLECULE_LOCALIZATION	136	1.871E-05	CD74,ABCG1
	ADAPTIVE_IMMUNE_RESPONSE_GO_0002460	16	1.963E-05	CD74,C2
	ORGAN_MORPHOGENESIS	101	1.964E-05	CCL2,COL18A1
	RESPONSE_TO_OTHER_ORGANISM	47	2.08E-05	IRF7
	REGULATION_OF_CELL_GROWTH	27	2.892E-05	
	TRANSLATION	119	2.993E-05	
	EPITHELIAL_TO_MESENCHYMAL_TRANSITION	10	3.54E-05	S100A4
	LYMPHOCYTE_ACTIVATION	35	3.547E-05	
	MYELOID_CELL_DIFFERENTIATION	30	3.808E-05	
	PROTEIN_TRANSPORT	102	3.943E-05	CD74,ABCG1
	PROTEIN_LOCALIZATION	125	4.049E-05	CD74,ABCG1
	STRUCTURAL_CONSTITUENT_OF_CYTOSKELETON	26	4.061E-05	
	REGULATION_OF_RNA_METABOLIC_PROCESS	283	4.133E-05	CREM,FST,IRF7
Down (120hr)	MITOCHONDRION	217	2.864E-27	GATM,DMGDH
	ORGANIC_ACID_METABOLIC_PROCESS	130	1.756E-20	GATM,PAH,FTCD
	CARBOXYLIC_ACID_METABOLIC_PROCESS	128	3.293E-19	GATM,PAH,FTCD
	AMINO_ACID_AND_DERIVATIVE_METABOLIC_PROCESS	80	1.137E-17	GATM,PAH
	ANION_CATION_SYMPORTER_ACTIVITY	11	2.463E-16	SLC12A3,SLC34A3,SLC13A2,SLC4A4
	SYMPORTER_ACTIVITY	26	9.921E-16	SLC5A2,SLC4A4,SLC12A3,SLC34A3,SLC13A2
	AMINO_ACID_METABOLIC_PROCESS	61	1.704E-15	PAH
	SECONDARY_ACTIVE_TRANSMEMBRANE_TRANSPORTER_ACTIVITY	35	2.376E-15	SLC5A2,SLC4A4,SLC12A3,SLC34A3,SLC13A2
	OXIDOREDUCTASE_ACTIVITY	203	9.888E-15	ALDH8A1,MIOX,PAH,DMGDH,NOX4
	NITROGEN_COMPOUND_METABOLIC_PROCESS	115	1.514E-14	GATM,PAH
	AMINE_METABOLIC_PROCESS	107	1.911E-14	GATM,PAH
	ANION_TRANSMEMBRANE_TRANSPORTER_ACTIVITY	42	9.409E-13	SLC22A6,SLC4A4,SLC22A8,SLC34A3,SLC12A3,SLC13A2
	SUBSTRATE_SPECIFIC_TRANSMEMBRANE_TRANSPORTER_ACTIVITY	277	3.952E-12	SLC22A6,SLC22A2,SLC22A8,CACNG5,SLC4A4,SLC34A3,SLC13A2,SLC5A2,SLC12A3
	MITOCHONDRIAL_PART	100	7.041E-11	GATM
	LYASE_ACTIVITY	53	1.687E-10	CA3,ACMSD,CBS
	ION_TRANSMEMBRANE_TRANSPORTER_ACTIVITY	224	2.284E-09	SLC22A6,SLC22A2,SLC22A8,CACNG5,SLC4A4,SLC34A3,SLC13A2,SLC5A2,SLC12A3
	TRANSMEMBRANE_TRANSPORTER_ACTIVITY	301	2.303E-09	SLC22A6,SLC22A2,SLC22A8,CACNG5,SLC4A4,SLC34A3,SLC13A2,SLC5A2,SLC12A3
	NITROGEN_COMPOUND_CATABOLIC_PROCESS	26	3.471E-09	
	COFACTOR_METABOLIC_PROCESS	36	2.023E-08	UGT1A1,FTCD
	ACTIVE_TRANSMEMBRANE_TRANSPORTER_ACTIVITY	98	2.184E-08	SLC4A4,SLC34A3,SLC13A2,SLC5A2,SLC12A3
	AMINO_ACID_CATABOLIC_PROCESS	22	5.27E-08	
	CATION_TRANSMEMBRANE_TRANSPORTER_ACTIVITY	175	6.249E-08	SLC22A2,CACNG5,SLC4A4,SLC34A3,SLC13A2,SLC5A2,SLC12A3
	HYDRO_LYASE_ACTIVITY	17	7.347E-08	CA3,CBS
	SUBSTRATE_SPECIFIC_TRANSPORTER_ACTIVITY	304	7.575E-08	SLC22A6,SLC22A2,SLC22A8,CACNG5,SLC34A3,SLC13A2,SLC12A3,SLC4A4,SLC5A2
	ANION_TRANSPORT	23	1.003E-07	SLC22A6,SLC22A8,SLC34A3
	AMINE_CATABOLIC_PROCESS	24	1.535E-07	
	GENERATION_OF_PRECURSOR_METABOLITES_AND_ENERGY	92	2.253E-07	NOX4
	CARBOHYDRATE_TRANSMEMBRANE_TRANSPORTER_ACTIVITY	14	2.451E-07	SLC5A2
	MITOCHONDRIAL_ENVELOPE	69	2.905E-07	GATM
	CARBON_OXYGEN_LYASE_ACTIVITY	21	4.489E-07	CA3,CBS
	ELECTRON_CARRIER_ACTIVITY	55	6.846E-07	DMGDH,NOX4
	INORGANIC_ANION_TRANSMEMBRANE_TRANSPORTER_ACTIVITY	16	7.508E-07	SLC34A3,SLC4A4
	CELLULAR_CATABOLIC_PROCESS	157	9.64E-07	GBA3,MIOX,UGT1A1,UGT2B4
	OXIDOREDUCTASE_ACTIVITY__ACTING_ON_THE_CH_CH_GROUP_OF_DONORS	19	1.049E-06	
	AROMATIC_COMPOUND_METABOLIC_PROCESS	20	1.441E-06	FTCD
	SUGAR_TRANSMEMBRANE_TRANSPORTER_ACTIVITY	11	1.589E-06	SLC5A2
	MICROBODY	34	1.966E-06	PEX11A
	PEROXISOME	34	1.966E-06	PEX11A
	ORGANIC_ACID_TRANSPORT	31	2.099E-06	SLC22A6
	CATABOLIC_PROCESS	165	2.675E-06	GBA3,MIOX,UGT1A1,UGT2B4

COENZYME_METABOLIC_PROCESS	24	6.3E-06	FTCD
COFACTOR_BINDING	17	6.735E-06	NOX4
CARBOXYLIC_ACID_TRANSPORT	30	1.55E-05	SLC22A6
ORGANIC_ACID_TRANSMEMBRANE_TRANSPORTER_ACTIVITY	35	1.807E-05	SLC13A2
LIPID_CATABOLIC_PROCESS	25	2.093E-05	UGT2B4
MITOCHONDRIAL_MATRIX	32	2.682E-05	
MITOCHONDRIAL_LUMEN	32	2.682E-05	
CELLULAR_LIPID_CATABOLIC_PROCESS	24	3.322E-05	UGT2B4
