# Supplementary material for: Gene Expression Analysis Reveals the Cell Cycle and Kinetochore Genes Participating in Ischemia Reperfusion Injury and Early Development in Kidney
Source: PLoS One. 2011 Sep 28;6(9):e25679. doi: 10.1371/journal.pone.0025679 (PMC3181346; doi:10.1371/journal.pone.0025679)
Supplement: Table S3 — The GO functional categories significantly enriched in developing kidney. The expression profiles from postnatal day 1 and 7 (PN1d and PN7d) are compared to that of embryonic kidney (E16d). (PDF) [file pone.0025679.s004.pdf]

Supplementary Table 3. The GO functional categories significantly enriched in developing kidney. The expression profiles from postnatal day 1 and 7 (PN1d and PN7d) are compared to that of embryonic kidney (E16d).

Scale	GO category	Gene	Pvalue	Leading edge genes
PN1d (up)	SUBSTRATE_SPECIFIC_TRANSPORTER_ACTIVITY	329	8.36E-24	SLC22A6,SLC3A1,AQP2,KCNJ15
	ORGANIC_ACID_METABOLIC_PROCESS	142	3.215E-23	FAH,SLC7A7,PAH,NR1H4,HGD,ACOX2,SLC3A1
	EXCRETION	35	3.788E-23	AQP2,GUCA2B
	OXIDOREDUCTASE_ACTIVITY	212	6.985E-23	CYP24A1,HGD,GPX3,PAH,ACOX2,HMOX1,ABP1
	TRANSMEMBRANE_TRANSPORTER_ACTIVITY	319	7.526E-23	AQP2,KCNJ15,SLC22A6,SLC3A1
	CARBOXYLIC_ACID_METABOLIC_PROCESS	140	1.063E-21	FAH,SLC7A7,PAH,NR1H4,HGD,ACOX2,SLC3A1
	SUBSTRATE_SPECIFIC_TRANSMEMBRANE_TRANSPORTER_ACTIVITY	294	6.649E-21	KCNJ15,SLC22A6,SLC3A1
	AMINO_ACID_METABOLIC_PROCESS	69	1.574E-20	SLC7A7,FAH,SLC3A1,PAH,HGD
	NITROGEN_COMPOUND_METABOLIC_PROCESS	125	1.635E-20	FAH,SLC7A7,ASS1,PAH,HGD,SLC3A1,SULT1C2
	PLASMA_MEMBRANE_PART	927	1.021E-17	SLC22A6,SLC3A1,TREH,TACSTD2,KCNJ15,ANPEP,SLC7A7,AQP2
	AMINO_ACID_AND_DERIVATIVE_METABOLIC_PROCESS	87	4.506E-17	FAH,SLC7A7,PAH,HGD,SLC3A1
	AMINE_METABOLIC_PROCESS	116	9.676E-17	FAH,SLC7A7,PAH,HGD,SLC3A1,SULT1C2
	ANION_TRANSMEMBRANE_TRANSPORTER_ACTIVITY	50	6.566E-16	SLC22A6
	INTRINSIC_TO_PLASMA_MEMBRANE	804	9.012E-16	SLC22A6,SLC3A1,TREH,TACSTD2,KCNJ15,ANPEP,SLC7A7
	MEMBRANE_FRACTION	257	1.79E-15	SLC22A6,SLC3A1,HMOX1
	INTEGRAL_TO_PLASMA_MEMBRANE	792	2.285E-15	SLC22A6,SLC3A1,TACSTD2,KCNJ15,ANPEP,SLC7A7
	ACTIVE_TRANSMEMBRANE_TRANSPORTER_ACTIVITY	105	7.318E-15	SLC3A1
	ION_TRANSMEMBRANE_TRANSPORTER_ACTIVITY	235	1.087E-14	KCNJ15,SLC22A6
	ESTABLISHMENT_OF_LOCALIZATION	663	1.318E-14	SLC22A6,SLC3A1,KCNJ15,GUCA2B,SLC7A7,AQP2
	CELL_FRACTION	371	3.055E-13	SLC22A6,SLC3A1,HMOX1
	NITROGEN_COMPOUND_CATABOLIC_PROCESS	26	5.429E-13	FAH,HGD
	TRANSPORT	609	1.198E-11	SLC22A6,SLC3A1,KCNJ15,SLC7A7,AQP2
	ORGANIC_ACID_TRANSPORT	35	1.271E-11	SLC22A6,SLC3A1
	CARBOXYLIC_ACID_TRANSPORT	34	1.328E-11	SLC22A6,SLC3A1
	GENERATION_OF_PRECURSOR_METABOLITES_AND_ENERGY	98	1.448E-11	CYP24A1,TREH
	AMINO_ACID_CATABOLIC_PROCESS	23	2.405E-11	FAH,HGD
	PROTEIN_TETRAMERIZATION	10	6.362E-11	GPX3
	SECRETION	129	8.365E-11	AQP2,GUCA2B
	AMINE_CATABOLIC_PROCESS	24	9.168E-11	FAH,HGD
	EXTRACELLULAR_REGION	353	9.581E-11	FGA,KLK6,GPX3
	SYMPORTER_ACTIVITY	30	1.459E-10	
	EXTRACELLULAR_SPACE	184	1.679E-10	GPX3,FGA
	OXIDOREDUCTASE_ACTIVITY__ACTING_ON_PEROXIDE_AS_ACCEPTOR	11	2.277E-10	GPX3
	SECONDARY_ACTIVE_TRANSMEMBRANE_TRANSPORTER_ACTIVITY	42	4.585E-10	
	PROTEASE_INHIBITOR_ACTIVITY	30	8.601E-10	SERPINA10,SERPINA6
	ANION_CATION_SYMPORTER_ACTIVITY	15	1.515E-09	
	CATION_TRANSMEMBRANE_TRANSPORTER_ACTIVITY	183	1.913E-09	KCNJ15
	INORGANIC_ANION_TRANSMEMBRANE_TRANSPORTER_ACTIVITY	17	4.313E-09	
	ORGANIC_ACID_TRANSMEMBRANE_TRANSPORTER_ACTIVITY	40	6.19E-09	SLC3A1
	CARBOXYLIC_ACID_TRANSMEMBRANE_TRANSPORTER_ACTIVITY	39	7.148E-09	SLC3A1
	EXTRACELLULAR_REGION_PART	265	8.686E-09	GPX3,FGA
	OXYGEN_AND_REACTIVE_OXYGEN_SPECIES_METABOLIC_PROCESS	15	1.036E-08	GPX3
	ANTIOXIDANT_ACTIVITY	16	1.136E-08	GPX3
	GLUTATHIONE_TRANSFERASE_ACTIVITY	15	1.238E-08	GSTA2
	CELLULAR_CARBOHYDRATE_METABOLIC_PROCESS	95	2.58E-08	FBP1,TREH,PDK4
	PHOSPHATE_TRANSMEMBRANE_TRANSPORTER_ACTIVITY	11	2.951E-08	
	ELECTRON_TRANSPORT_GO_0006118	37	7.946E-08	CYP24A1
	CARBOHYDRATE_METABOLIC_PROCESS	131	1.06E-07	FBP1,TREH,PDK4
	LIPID_METABOLIC_PROCESS	231	1.274E-07	NR1H4,ACOX2
	SERINE_TYPE_ENDOPEPTIDASE_INHIBITOR_ACTIVITY	19	1.423E-07	SERPINA10,SERPINA6
	L_AMINO_ACID_TRANSMEMBRANE_TRANSPORTER_ACTIVITY	15	1.552E-07	SLC3A1
	AMINO_ACID_TRANSPORT	22	3.097E-07	SLC3A1
	SYSTEM_PROCESS	458	3.254E-07	GUCA2B,AQP2,TACSTD2
	PROTEIN_OLIGOMERIZATION	24	4.554E-07	GPX3
	MITOCHONDRION	251	5.395E-07	PDK4
	CARBON_CARBON_LYASE_ACTIVITY	17	6.198E-07	PCK1
	ION_TRANSPORT	150	6.518E-07	KCNJ15,SLC22A6
	RESPONSE_TO_WOUNDING	158	9.119E-07	S100A8,S100A9
	RESPONSE_TO_OXIDATIVE_STRESS	36	1.128E-06	GPX3
	APICAL_PART_OF_CELL	11	1.138E-06	AQP2
	ANION_TRANSPORT	23	1.871E-06	SLC22A6
	COENZYME_METABOLIC_PROCESS	25	1.891E-06	GSTA1
	GLUCOSE_METABOLIC_PROCESS	21	2.046E-06	PDK4
	DEFENSE_RESPONSE	191	2.605E-06	S100A8,S100A9,UMOD
	INFLAMMATORY_RESPONSE	105	2.964E-06	S100A8,S100A9
	PROTEIN_HOMOOLIGOMERIZATION	15	4.05E-06	GPX3
	RESPONSE_TO_EXTERNAL_STIMULUS	258	4.545E-06	S100A8,S100A9
	BODY_FLUID_SECRETION	10	5.385E-06	
	HYDROLASE_ACTIVITY__HYDROLYZING_O_GLYCOSYL_COMPOUNDS	25	1.084E-05	TREH
	REGULATION_OF_BODY_FLUID_LEVELS	48	1.088E-05	
	MICROBODY_PART	11	1.196E-05	
	PEROXISOMAL_PART	11	1.196E-05	

	CELLULAR_LIPID_METABOLIC_PROCESS	181	1.378E-05	NR1H4,ACOX2
	ALCOHOL_METABOLIC_PROCESS	69	1.732E-05	FBP1,PDK4
	AMINO_ACID_TRANSMEMBRANE_TRANSPORTER_ACTIVITY	25	1.765E-05	SLC3A1
	APICAL_PLASMA_MEMBRANE	10	2.322E-05	AQP2
	PEPTIDASE_ACTIVITY	135	2.712E-05	ANPEP,DPEP1
	BILE_ACID_METABOLIC_PROCESS	10	3.143E-05	ACOX2,NR1H4
	AMINE_TRANSPORT	31	3.658E-05	SLC3A1
PN1d (down)	NUCLEUS	982	7.662E-48	
	NUCLEAR_PART	377	7.463E-38	
	NUCLEOBASE__NUCLEOSIDE__NUCLEOTIDE_AND_NUCLEIC_ACID_METABOLIC_PROCESS	909	9.317E-36	
	INTRACELLULAR_ORGANELLE_PART	817	2.015E-24	ALAS2
	ORGANELLE_PART	821	8.507E-24	ALAS2
	RNA_METABOLIC_PROCESS	594	1.669E-22	
	NUCLEAR_LUMEN	240	4.725E-20	
	MACROMOLECULAR_COMPLEX	683	1.954E-19	
	CELL_CYCLE_GO_0007049	245	2.049E-18	
	NUCLEOPLASM	181	4.237E-17	
	DNA_METABOLIC_PROCESS	204	1.172E-16	
	RNA_PROCESSING	113	1.227E-16	
	NON_MEMBRANE_BOUND_ORGANELLE	419	8.036E-16	
	INTRACELLULAR_NON_MEMBRANE_BOUND_ORGANELLE	419	8.036E-16	
	DNA_REPLICATION	79	9.268E-16	
	RIBONUCLEOPROTEIN_COMPLEX	101	1.224E-15	
	CELL_CYCLE_PROCESS	147	2.375E-15	
	RNA_SPLICING	62	3.315E-15	
	ORGANELLE_LUMEN	297	5.67E-15	
	MEMBRANE_ENCLOSED_LUMEN	297	5.67E-15	
	MITOTIC_CELL_CYCLE	115	2.824E-14	
	SPLICEOSOME	31	2.363E-13	
	CELL_CYCLE_PHASE	131	1.002E-12	
	MRNA_METABOLIC_PROCESS	59	1.991E-12	
	NUCLEOPLASM_PART	136	2.139E-12	
	DNA_BINDING	448	6.928E-12	
	MRNA_PROCESSING_GO_0006397	52	7.534E-12	
	CHROMOSOME	89	8.247E-12	
	M_PHASE	86	2.887E-11	
	RNA_BINDING	188	3.975E-11	
	PROTEIN_COMPLEX	595	5.631E-11	
	RNA_BIOSYNTHETIC_PROCESS	459	1.232E-10	
	RESPONSE_TO_DNA_DAMAGE_STIMULUS	121	1.768E-10	
	TRANSCRIPTION	537	2.122E-10	
	DNA_DEPENDENT_DNA_REPLICATION	44	2.61E-10	
	M_PHASE_OF_MITOTIC_CELL_CYCLE	62	3.226E-10	
	TRANSCRIPTION__DNA_DEPENDENT	457	3.443E-10	
	MITOSIS	60	4.329E-10	
	DNA_REPAIR	99	7.107E-10	
	REGULATION_OF_CELL_CYCLE	143	7.231E-10	
	CHROMOSOME_ORGANIZATION_AND_BIOGENESIS	89	2.259E-09	
	REGULATION_OF_NUCLEOBASE__NUCLEOSIDE__NUCLEOTIDE_AND_NUCLEIC_ACID_METABOLIC_PROCESS	432	5.361E-09	
	CHROMOSOMAL_PART	68	5.974E-09	
	RESPONSE_TO_ENDOGENOUS_STIMULUS	150	3.279E-08	
	SPINDLE	29	3.445E-08	
	REGULATION_OF_RNA_METABOLIC_PROCESS	326	8.047E-08	
	RIBONUCLEOPROTEIN_COMPLEX_BIOGENESIS_AND_ASSEMBLY	56	8.123E-08	
	TRANSCRIPTION_FROM_RNA_POLYMERASE_II_PROMOTER	328	8.396E-08	
	SMALL_NUCLEAR_RIBONUCLEOPROTEIN_COMPLEX	16	1.086E-07	
	MICROTUBULE_CYTOSKELETON	98	1.478E-07	
	NUCLEAR_MEMBRANE_PART	32	1.587E-07	
	REGULATION_OF_CELLULAR_METABOLIC_PROCESS	548	2.283E-07	
	NUCLEAR_MEMBRANE	40	3.358E-07	
	REGULATION_OF_METABOLIC_PROCESS	557	3.379E-07	
	REGULATION_OF_TRANSCRIPTION__DNA_DEPENDENT	320	3.405E-07	
	REGULATION_OF_MITOSIS	33	4.803E-07	
	ESTABLISHMENT_AND_OR_MAINTENANCE_OF_CHROMATIN_ARCHITECTURE	60	5.2E-07	
	NUCLEAR_PORE	25	5.45E-07	
	CHROMATIN_BINDING	25	5.672E-07	
	REGULATION_OF_TRANSCRIPTION	393	6.22E-07	
	NUCLEAR_ENVELOPE	59	7.545E-07	
	STRUCTURE_SPECIFIC_DNA_BINDING	46	8.774E-07	
	RNA_SPLICING__VIA_TRANSESTERIFICATION_REACTIONS	27	1.072E-06	
	NUCLEOLUS	75	1.264E-06	
	CELL_CYCLE_CHECKPOINT_GO_0000075	36	1.681E-06	
	PORE_COMPLEX	29	2.591E-06	
	REGULATION_OF_GENE_EXPRESSION	467	2.861E-06	
	CENTROSOME	34	3.329E-06	
	INTERPHASE_OF_MITOTIC_CELL_CYCLE	51	3.875E-06	
	REPLICATION_FORK	13	3.966E-06	
	MICROTUBULE_ORGANIZING_CENTER	41	4.257E-06	
	CYTOSKELETON	242	4.899E-06	
	CYTOSKELETAL_PART	148	7.574E-06	
	CHROMOSOME__PERICENTRIC_REGION	23	8.229E-06	
	INTERPHASE	55	9.54E-06	
	DNA_RECOMBINATION	42	9.861E-06	
	TRANSCRIPTION_FACTOR_COMPLEX	67	1.107E-05	
	SPINDLE_MICROTUBULE	13	1.242E-05	
	PROTEIN_DNA_COMPLEX_ASSEMBLY	33	2.158E-05	
	TRANSCRIPTION_COFACTOR_ACTIVITY	164	2.805E-05	
	CHROMATIN_REMODELING_COMPLEX	13	2.928E-05	

	NUCLEOCYTOPLASMIC_TRANSPORT	67	2.955E-05	
	NUCLEAR_TRANSPORT	68	3.885E-05	
PN7d (up)	TRANSMEMBRANE_TRANSPORTER_ACTIVITY	319	1.581E-26	KCNJ1,KCNJ15,SLC22A1,SLC22A8,SLC26A4,BSND,SLC7A8,SLC7A9,SLC34A1,SLC25A10,SLC13A2,SLC5A2,SLC1A1,CLCNKB,SLC12A3,SLC14A2
	SUBSTRATE_SPECIFIC_TRANSPORTER_ACTIVITY	329	3.689E-26	KCNJ1,SLC22A1,SLC22A8,SLC7A8,SLC7A9,SLC34A1,SLC13A2,SLC1A1,CLCNKB,SLC12A3,SLC14A2,AQP1,APOM,KCNJ15,SLC26A4,BSND,SLC25A10,SLC5A2
	SUBSTRATE_SPECIFIC_TRANSMEMBRANE_TRANSPORTER_ACTIVITY	294	1.836E-25	KCNJ1,KCNJ15,SLC22A1,SLC22A8,SLC26A4,BSND,SLC7A8,SLC7A9,SLC34A1,SLC25A10,SLC13A2,SLC5A2,SLC1A1,CLCNKB,SLC12A3,SLC14A2
	EXCRETION	35	9.595E-23	CLCNKB,AQP1,KCNJ1,KNG1
	ANION_TRANSMEMBRANE_TRANSPORTER_ACTIVITY	50	2.948E-20	SLC22A8,SLC34A1,SLC26A4,BSND,CLCNKB,SLC12A3,SLC13A2
	ION_TRANSMEMBRANE_TRANSPORTER_ACTIVITY	235	1.417E-19	KCNJ1,KCNJ15,SLC22A1,SLC22A8,SLC26A4,BSND,SLC34A1,SLC13A2,SLC5A2,CLCNKB,SLC12A3
	ACTIVE_TRANSMEMBRANE_TRANSPORTER_ACTIVITY	105	2.676E-16	SLC7A8,SLC7A9,SLC13A2,SLC5A2,SLC1A1,SLC12A3
	PLASMA_MEMBRANE_PART	927	1.159E-15	AGTR1,ATP12A,SLC22A1,SLC22A8,SLC13A2,SLC1A1,CLDN2,CLCNKB,KL,MEP1A,APOM,KCNJ15,BSND,KCNJ1,SLC7A8,SLC7A9,SLC34A1,SLC12A3,AQP1
	INTEGRAL_TO_PLASMA_MEMBRANE	792	5.012E-15	AGTR1,ATP12A,SLC22A1,SLC22A8,SLC13A2,SLC1A1,CLCNKB,KL,MEP1A,APOM,KCNJ15,BSND,KCNJ1,SLC7A8,SLC7A9,SLC34A1,SLC12A3,AQP1
	INTRINSIC_TO_PLASMA_MEMBRANE	804	7.701E-15	AGTR1,ATP12A,SLC22A1,SLC22A8,SLC13A2,SLC1A1,CLCNKB,KL,MEP1A,APOM,KCNJ15,BSND,KCNJ1,SLC7A8,SLC7A9,SLC34A1,SLC12A3,AQP1
	SECONDARY_ACTIVE_TRANSMEMBRANE_TRANSPORTER_ACTIVITY	42	4.379E-14	SLC5A2,SLC12A3,SLC13A2
	SYMPORTER_ACTIVITY	30	1.38E-13	SLC5A2,SLC12A3,SLC13A2
	ORGANIC_ACID_METABOLIC_PROCESS	142	1.824E-13	SLC7A8,SLC7A9,FAH,PAH,NR1H4,HGD,SLC25A10,PTGDS,SLC27A2
	CATION_TRANSMEMBRANE_TRANSPORTER_ACTIVITY	183	1.956E-13	KCNJ1,KCNJ15,SLC22A1,SLC13A2,SLC5A2,SLC12A3
	CARBOXYLIC_ACID_METABOLIC_PROCESS	140	2.675E-13	SLC7A8,SLC7A9,FAH,PAH,NR1H4,HGD,SLC25A10,PTGDS,SLC27A2
	MEMBRANE_FRACTION	257	3.513E-13	SLC22A1,SLC22A8,SLC13A2,SLC1A1,KL,SLC12A3,SLC14A2
	ION_TRANSPORT	150	1.227E-12	SLC34A1,KCNJ1,UCP1,KCNJ15,SLC22A1,SLC22A8,SLC26A4
	EXTRACELLULAR_REGION	353	2.038E-12	C2,KLK6,KL,KLK5,MEP1A,PRELP,CDA,SFRP1,GPX3,PTGDS,TCN2,TINAG,IGFALS
	NITROGEN_COMPOUND_METABOLIC_PROCESS	125	3.528E-12	SLC7A8,SLC7A9,FAH,ASS1,PAH,HGD,SULT1C2
	ESTABLISHMENT_OF_LOCALIZATION	663	1.027E-11	SLC22A1,SLC22A8,CUBN,SLC1A1,CLCNKB,CIDEA,KCNJ15,SLC26A4,PTGDS,TCN2,SLC25A10,CRYAB,KCNJ1,SLC7A8,SLC7A9,SLC34A1,KNG1,SLC14A2,AQP1,UCP1
	GENERATION_OF_PRECURSOR_METABOLITES_AND_ENERGY	98	3.139E-11	APOM,XYLB,NOX4
	SECRETION	129	9.254E-11	AQP1,SLC34A1,KCNJ1,CIDEA,KNG1,CLCNKB
	AMINE_METABOLIC_PROCESS	116	9.63E-11	SLC7A8,SLC7A9,FAH,PAH,HGD,SULT1C2
	OXYGEN_AND_REACTIVE_OXYGEN_SPECIES_METABOLIC_PROCESS	15	4.142E-10	NOX4,GPX3
	PROTEIN_TETRAMERIZATION	10	7.229E-10	GPX3,CDA
	CELL_FRACTION	371	1.037E-09	SLC22A1,SLC22A8,SLC13A2,SLC1A1,KL,SLC12A3,MEP1A,SLC14A2,IGFALS
	ANION_CATION_SYMPORTER_ACTIVITY	15	1.227E-09	SLC12A3,SLC13A2
	OXIDOREDUCTASE_ACTIVITY	212	1.288E-09	EHHADH,HGD,GPX3,PAH,NOX4
	TRANSPORT	609	2.593E-09	SLC22A1,SLC22A8,CUBN,SLC1A1,KCNJ15,SLC26A4,PTGDS,TCN2,SLC25A10,CRYAB,KCNJ1,SLC7A8,SLC7A9,SLC34A1,SLC14A2,AQP1,UCP1
	AMINO_ACID_METABOLIC_PROCESS	69	3.244E-09	SLC7A8,SLC7A9,FAH,PAH,HGD
	EXTRACELLULAR_REGION_PART	265	3.618E-09	PRELP,SFRP1,GPX3,C2,TCN2,TINAG,IGFALS,KL,KLK5,MEP1A
	ANION_TRANSPORT	23	3.784E-09	SLC22A8,SLC34A1,SLC26A4
	AMINO_ACID_AND_DERIVATIVE_METABOLIC_PROCESS	87	5.514E-09	SLC7A8,SLC7A9,FAH,PAH,HGD
	CATION_TRANSPORT	121	5.604E-09	KCNJ1,UCP1,KCNJ15,SLC22A1
	SYSTEM_PROCESS	458	5.755E-09	CRYAB,KCNJ1,SLC1A1,KNG1,CLCNKB,AQP1,SLC26A4
	LIPID_METABOLIC_PROCESS	231	1.393E-08	APOM,NR1H4,CIDEA,PTGDS,SLC27A2
	INORGANIC_ANION_TRANSMEMBRANE_TRANSPORTER_ACTIVITY	17	3.123E-08	SLC34A1,SLC26A4
	CARBOHYDRATE_METABOLIC_PROCESS	131	4.327E-08	FBP1,SLC25A10,SLC5A2,XYLB,PDK4
	GLUTATHIONE_TRANSFERASE_ACTIVITY	15	5.494E-08	GSTT2
	SOLUTE_SODIUM_SYMPORTER_ACTIVITY	13	8.011E-08	SLC5A2,SLC13A2
	MICROBODY	35	1.294E-07	EHHADH,SLC27A2
	PEROXISOME	35	1.294E-07	EHHADH,SLC27A2
	ORGANIC_ACID_TRANSPORT	35	1.605E-07	SLC7A8,SLC7A9,SLC1A1,SLC25A10
	ORGANIC_ACID_TRANSMEMBRANE_TRANSPORTER_ACTIVITY	40	1.649E-07	SLC7A8,SLC7A9,SLC1A1,SLC25A10,SLC13A2
	CARBOXYLIC_ACID_TRANSPORT	34	3.436E-07	SLC7A8,SLC7A9,SLC1A1,SLC25A10
	CARBOXYLIC_ACID_TRANSMEMBRANE_TRANSPORTER_ACTIVITY	39	3.44E-07	SLC7A8,SLC7A9,SLC1A1,SLC25A10,SLC13A2
	OXIDOREDUCTASE_ACTIVITY__ACTING_ON_PEROXIDE_AS_ACCEPTOR	11	6.777E-07	GPX3

	PROTEIN_OLIGOMERIZATION	24	1.254E-06	GPX3,CDA
	MONOVALENT_INORGANIC_CATION_TRANSPORT	74	1.832E-06	KCNJ15,KCNJ1,UCP1
	COENZYME_BINDING	11	2.882E-06	NOX4
	CELLULAR_CARBOHYDRATE_METABOLIC_PROCESS	95	3.087E-06	FBP1,SLC25A10,PDK4
	EXTRACELLULAR_SPACE	184	3.188E-06	SFRP1,GPX3,C2,TCN2,IGFALS,KL,KLK5,MEP1A
	INORGANIC_ANION_TRANSPORT	14	3.342E-06	SLC34A1,SLC26A4
	PHOSPHATE_TRANSMEMBRANE_TRANSPORTER_ACTIVITY	11	4.334E-06	SLC34A1
	AMINO_ACID_CATABOLIC_PROCESS	23	5.241E-06	FAH,HGD
	CELLULAR_LIPID_METABOLIC_PROCESS	181	5.65E-06	APOM,NR1H4,PTGDS,SLC27A2
	APICAL_PART_OF_CELL	11	6.874E-06	
	MITOCHONDRION	251	7.495E-06	HMGCS2,PDK4
	NITROGEN_COMPOUND_CATABOLIC_PROCESS	26	8.294E-06	FAH,HGD
	RESPONSE_TO_EXTERNAL_STIMULUS	258	9.724E-06	F13B,PLAU,C2,KNG1,PROC,NOX4
	INORGANIC_CATION_TRANSMEMBRANE_TRANSPORTER_ACTIVITY	51	9.749E-06	
	L_AMINO_ACID_TRANSMEMBRANE_TRANSPORTER_ACTIVITY	15	1.017E-05	SLC7A9,SLC1A1
	AMINE_CATABOLIC_PROCESS	24	1.138E-05	FAH,HGD
	ELECTRON_TRANSPORT_GO_0006118	37	1.954E-05	NOX4
	PROTEASE_INHIBITOR_ACTIVITY	30	2.414E-05	SERPINA6,SPP2
	ANTIOXIDANT_ACTIVITY	16	2.476E-05	GPX3
	AMINO_ACID_TRANSPORT	22	2.582E-05	SLC7A8,SLC7A9,SLC1A1
PNd7 (down)	NUCLEUS	982	1.773E-62	KIF22,RANBP1,CDCA7,CDCA5,TPX2,CCNB1,H2AFX,CBX3,SOX9,MCM5,BUB1B,KPNA2,CENPA,APEX1
	NUCLEAR_PART	377	1.073E-48	BUB1B,KPNA2
	NUCLEOBASE__NUCLEOSIDE__NUCLEOTIDE_AND_NUCLEIC_ACID_METABOLIC_PROCESS	909	5.012E-42	NME4,FIGNL1,IGF2BP3,MCM5,KPNA2,APEX1
	INTRACELLULAR_ORGANELLE_PART	817	4.584E-34	KIF22,RPL13A,BIRC5,CDCA5,TPX2,PLK1,BUB1B,KPNA2,CENPA
	ORGANELLE_PART	821	1.259E-33	KIF22,RPL13A,BIRC5,CDCA5,TPX2,PLK1,BUB1B,KPNA2,CENPA
	RNA_METABOLIC_PROCESS	594	3.354E-29	IGF2BP3,APEX1
	MACROMOLECULAR_COMPLEX	683	1.832E-28	KIF22,RPL13A,GJA1,BIRC5,CDCA5,BUB1B,APEX1
	RNA_BINDING	188	2.724E-26	IGF2BP3,RPS24
	CELL_CYCLE_GO_0007049	245	6.089E-26	CDCA5,TPX2,BIRC5,STMN1,PLK1,KIF22,NUSAP1,BUB1B,KPNA2
	NON_MEMBRANE_BOUND_ORGANELLE	419	2.28E-25	CDCA5,TPX2,PLK1,KIF22,RPL13A,BIRC5,BUB1B,CENPA,APEX1
	INTRACELLULAR_NON_MEMBRANE_BOUND_ORGANELLE	419	2.28E-25	CDCA5,TPX2,PLK1,KIF22,RPL13A,BIRC5,BUB1B,CENPA,APEX1
	RNA_PROCESSING	113	4.601E-25	IGF2BP3
	CELL_CYCLE_PROCESS	147	3.14E-24	CDCA5,TPX2,BIRC5,STMN1,PLK1,KIF22,NUSAP1,BUB1B,KPNA2
	MITOTIC_CELL_CYCLE	115	2.775E-23	CDCA5,STMN1,PLK1,KIF22,TPX2,NUSAP1,BUB1B,KPNA2,BIRC5
	NUCLEAR_LUMEN	240	4.545E-22	KPNA2
	RIBONUCLEOPROTEIN_COMPLEX	101	1.521E-21	APEX1,RPL13A
	CHROMOSOME	89	2.33E-21	CDCA5,KIF22,BUB1B,CENPA,BIRC5
	CELL_CYCLE_PHASE	131	6.532E-20	CDCA5,PLK1,KIF22,TPX2,NUSAP1,BUB1B,KPNA2,BIRC5
	RNA_SPLICING	62	5.084E-19	
	DNA_METABOLIC_PROCESS	204	5.465E-19	MCM5,KPNA2,APEX1
	M_PHASE	86	1.706E-17	CDCA5,PLK1,KIF22,TPX2,NUSAP1,BUB1B,KPNA2,BIRC5
	DNA_REPLICATION	79	3.443E-17	MCM5
	M_PHASE_OF_MITOTIC_CELL_CYCLE	62	5.471E-17	KIF22,CDCA5,TPX2,NUSAP1,BUB1B,BIRC5,PLK1
	ORGANELLE_LUMEN	297	7.095E-17	KPNA2
	MEMBRANE_ENCLOSED_LUMEN	297	7.095E-17	KPNA2
	CHROMOSOMAL_PART	68	2.4E-16	KIF22,CDCA5,BUB1B,CENPA,BIRC5
	SPLICEOSOME	31	2.654E-16	
	MITOSIS	60	4.137E-16	KIF22,CDCA5,TPX2,NUSAP1,BUB1B,BIRC5,PLK1
	PROTEIN_COMPLEX	595	6.326E-16	KIF22,GJA1,BIRC5,CDCA5,BUB1B
	NUCLEOPLASM	181	8.886E-16	KPNA2
	MRNA_PROCESSING_GO_0006397	52	3.716E-14	
	MRNA_METABOLIC_PROCESS	59	5.038E-14	
	DNA_BINDING	448	6.442E-14	HEY1,APEX1
	CHROMATIN_BINDING	25	7.239E-13	CBX3,CENPA,CDCA5
	RIBONUCLEOPROTEIN_COMPLEX_BIOGENESIS_AND_ASSEMBLY	56	3.028E-12	
	CHROMOSOME_ORGANIZATION_AND_BIOGENESIS	89	3.228E-12	CDCA5,NUSAP1
	TRANSCRIPTION	537	3.808E-12	APEX1
	RNA_BIOSYNTHETIC_PROCESS	459	6.309E-12	APEX1
	CHROMOSOME__PERICENTRIC_REGION	23	6.45E-12	KIF22,CENPA,BIRC5,BUB1B
	NUCLEAR_ENVELOPE	59	9.081E-12	
	DNA_DEPENDENT_DNA_REPLICATION	44	1.515E-11	
	TRANSCRIPTION__DNA_DEPENDENT	457	2.318E-11	APEX1
	NUCLEOLUS	75	2.369E-11	
	NUCLEAR_MEMBRANE_PART	32	2.775E-11	
	REGULATION_OF_CELL_CYCLE	143	4.043E-11	NUSAP1,BUB1B,BIRC5
	SPINDLE	29	5.723E-11	TPX2,BIRC5
	NUCLEAR_MEMBRANE	40	9.262E-11	
	REGULATION_OF_NUCLEOBASE__NUCLEOSIDE__NUCLEOTIDE_AND_NUCLEIC_ACID_METABOLIC_PROCESS	432	1.822E-10	KPNA2
	SMALL_NUCLEAR_RIBONUCLEOPROTEIN_COMPLEX	16	2.271E-10	
	NUCLEAR_PORE	25	2.692E-10	
	DNA_REPAIR	99	3.557E-10	APEX1
	REGULATION_OF_MITOSIS	33	9.069E-10	NUSAP1,BIRC5,BUB1B
	NUCLEOPLASM_PART	136	1.333E-09	
	STRUCTURAL_CONSTITUENT_OF_RIBOSOME	73	1.692E-09	RPS24,RPL13A

PORE_COMPLEX	29	3.577E-09	
RESPONSE_TO_DNA_DAMAGE_STIMULUS	121	4.506E-09	APEX1
REGULATION_OF_GENE_EXPRESSION	467	4.775E-09	
MICROTUBULE_CYTOSKELETON	98	5.037E-09	PLK1,TPX2,BIRC5
RNA_SPLICING__VIA_TRANSESTERIFICATION_REACTIONS	27	7.709E-09	
INTERPHASE_OF_MITOTIC_CELL_CYCLE	51	9.884E-09	CDCA5,KPNA2,BIRC5
RESPONSE_TO_ENDOGENOUS_STIMULUS	150	1.114E-08	APEX1
CYTOSKELETAL_PART	148	1.147E-08	TPX2,BIRC5,PLK1
INTERPHASE	55	1.195E-08	CDCA5,KPNA2,BIRC5
CYTOKINESIS	13	1.238E-08	NUSAP1,BIRC5
REGULATION_OF_RNA_METABOLIC_PROCESS	326	1.272E-08	
REGULATION_OF_CELLULAR_METABOLIC_PROCESS	548	1.605E-08	KPNA2
TRANSCRIPTION_FROM_RNA_POLYMERASE_II_PROMOTER	328	1.738E-08	APEX1
CELL_CYCLE_CHECKPOINT_GO_0000075	36	2.109E-08	BIRC5,BUB1B
REGULATION_OF_TRANSCRIPTION	393	3.813E-08	
REGULATION_OF_TRANSCRIPTION__DNA_DEPENDENT	320	4.584E-08	
REGULATION_OF_METABOLIC_PROCESS	557	5.782E-08	KPNA2
REPLICATION_FORK	13	9.947E-08	
CELL_DIVISION	15	1.113E-07	NUSAP1,BIRC5
ESTABLISHMENT_AND_OR_MAINTENANCE_OF_CHROMATIN_ARCHITECTURE	60	1.142E-07	
PROTEIN_RNA_COMPLEX_ASSEMBLY	43	2.188E-07	
CYTOSKELETON	242	2.391E-07	TPX2,BIRC5,PLK1
CENTROSOME	34	4.876E-07	BIRC5,PLK1
MICROTUBULE_CYTOSKELETON_ORGANIZATION_AND_BIOGENESIS	24	5.748E-07	NUSAP1,STMN1
CHROMOSOME_SEGREGATION	19	6.735E-07	CDCA5,NUSAP1
ORGANELLE_ORGANIZATION_AND_BIOGENESIS	347	7.396E-07	CDCA5,STMN1,NUSAP1,KPNA2
STRUCTURE_SPECIFIC_DNA_BINDING	46	9.311E-07	
DNA_PACKAGING	25	1.034E-06	CDCA5,NUSAP1
KINETOCHORE	19	1.239E-06	KIF22,BUB1B
TRANSLATION	133	1.343E-06	
ESTABLISHMENT_OF_ORGANELLE_LOCALIZATION	12	1.579E-06	NUSAP1,BIRC5,CDCA5
NUCLEAR_TRANSPORT	68	1.659E-06	KPNA2
MITOTIC_CELL_CYCLE_CHECKPOINT	17	2.196E-06	BUB1B
NUCLEOCYTOPLASMIC_TRANSPORT	67	2.683E-06	KPNA2
PROTEIN_DNA_COMPLEX_ASSEMBLY	33	2.702E-06	
TRANSCRIPTION_COFACTOR_ACTIVITY	164	3.23E-06	APEX1
DNA_RECOMBINATION	42	3.278E-06	KPNA2
TRANSLATION_REGULATOR_ACTIVITY	29	3.287E-06	
RRNA_PROCESSING	10	4.548E-06	
DNA_DEPENDENT_ATPASE_ACTIVITY	13	4.657E-06	
MICROTUBULE_ORGANIZING_CENTER	41	5.58E-06	BIRC5,PLK1
SPLICEOSOME_ASSEMBLY	17	8.756E-06	
REGULATION_OF_DNA_METABOLIC_PROCESS	36	9.863E-06	KPNA2
RNA_SPLICING_FACTOR_ACTIVITY__TRANSESTERIFICATION_MECHANISM	11	9.973E-06	
CHROMATIN_REMODELING_COMPLEX	13	1.003E-05	
SPINDLE_MICROTUBULE	13	1.015E-05	BIRC5
RIBOSOME_BIOGENESIS_AND_ASSEMBLY	12	1.09E-05	
RRNA_METABOLIC_PROCESS	11	1.101E-05	
TRANSLATION_FACTOR_ACTIVITY__NUCLEIC_ACID_BINDING	28	1.19E-05	
HELICASE_ACTIVITY	35	2.048E-05	
BASE_EXCISION_REPAIR	13	2.383E-05	APEX1
REGULATION_OF_DNA_REPLICATION	14	2.86E-05	
REGULATION_OF_TRANSCRIPTION_FROM_RNA_POLYMERASE_II_PROMOTER	195	3.144E-05	
ORGANELLE_LOCALIZATION	15	3.511E-05	CDCA5,NUSAP1,BIRC5
ENDOMEMBRANE_SYSTEM	161	3.647E-05	
